# Sensing nature in the city: The role of sight and sound in restorative tropical urban green spaces

**DOI:** 10.1371/journal.pone.0351647

**Published:** 2026-06-15

**Authors:** Juliana Ju Yun Hoo, Shumetha Sidhu, Kok Wei Tan

**Affiliations:** 1 School of Psychology and Clinical Language Sciences, University of Reading Malaysia, Iskandar Puteri, Malaysia; 2 School of Psychology and Clinical Language Sciences, University of Reading, Reading, Berkshire, United Kingdom; 3 Department of Social Science, Faculty of Social Science and Humanities, Tunku Abdul Rahman University of Management and Technology, Kuala Lumpur, Malaysia; East China Normal University, CHINA

## Abstract

Rapid urbanization has increased disconnection from nature, especially in cities. While research on restorative environments has largely focused on non-tropical regions, little is known about the restorative potential of tropical urban green spaces (UGSs). This study assessed the perceived restorativeness of tropical UGSs in Malaysia using 120 environmental stimuli from nature, urban, and mixed urban-nature settings. 87 participants were randomly assigned to one of the three modalities: audio-only, visual-only, or bimodal. Each participant rated a subset of 30 stimuli on perceived restorativeness. Results showed that nature and mixed urban-nature scenes were in general rated as more restorative than urban scenes. An interaction effect indicated that, in the visual-only modality, mixed urban-nature scenes were perceived as more restorative than nature scenes, while no significant differences were observed in the audio-only and bimodal modalities. Moreover, perceived restorativeness for nature scenes was comparable across bimodal, visual-only, and audio-only presentations. These findings suggest that small pockets of urban nature (e.g., tree-lined streets, rooftop gardens) can offer greater psychological restoration than wild, untamed forests. In addition, high-quality nature sounds (e.g., birdsong, flowing water) can provide restorative benefits comparable to visual exposure when access to green views is limited. Such insights can inform urban planning strategies to design more restorative and liveable cities.

## 1. Introduction

Urbanization is a global phenomenon, with 55% of the world’s population living in urban areas as of 2018—a figure projected to rise to 68% by 2050, with most growth occurring in Asia and Africa [1]. While cities offer numerous opportunities and conveniences, urban living is associated with higher levels of stress and mental health issues compared to rural living [2,3]. A growing body of research shows that natural environments offer restorative benefits for cognition [4–7], psychological well-being [8–11] and physiological health [12,13]. However, limited access to green spaces in cities may prevent urban residents from enjoying these benefits.

Urban green spaces (UGSs) play a crucial role in addressing this issue by providing accessible environments where people can reconnect with nature. According to the World Health Organization (WHO), UGSs include vegetated urban areas, such as parks, gardens, tree-lined streets, and nature reserves, as well as small water bodies like ponds, lakes, and streams [14]. Unlike general green spaces, which refer solely to vegetated areas, UGSs encompass a broader range of designed and managed urban environments intended for public use [15]. These spaces not only support biodiversity and provide opportunities for nature exposure but also contribute to the physical and mental well-being of urban residents [14]. Despite their importance, planning effective UGS remains challenging due to land scarcity, overdevelopment, and socio-economic disparities that result in unequal access of green spaces [16]. Understanding how urban dwellers perceive the restorativeness of different environmental settings is therefore essential for informing urban policy, planning, and design.

### 1.1. Restoration theories

As previously outlined, natural environments offer a wide range of benefits. Two prominent theories are used to explain the mechanisms through which these environments exert restorative effects: Attention Restoration Theory (ART) [17] and Stress Reduction Theory (SRT) [18]. ART proposed that prolonged demands on directed attention lead to mental fatigue, whereas environments that effortlessly capture attention through bottom-up processes help restore these attentional resources. Consistent with this account, exposure to nature has been linked to improved performance on tasks requiring sustained attention [6,19]. In contrast, SRT emphasizes affective and physiological pathways, suggesting that natural settings promote stress recovery by facilitating relaxation and regulating stress-related arousal [18,20]. Supporting evidence indicates that nature exposure is associated with greater positive affect and reduced negative affect [21], as well as physiological benefits such as lower heart rate and blood pressure [22]. In summary, ART highlights the cognitive benefits of nature by restoring depleted attentional resources, while SRT emphasizes nature’s role in alleviating physiological and psychological stress.

### 1.2. Perceived restorativeness in nature and urban settings

The concept of “perceived restorativeness” originates from Kaplan’s ART [17]. It refers to the extent to which an individual perceives an environment as restorative, that is, capable of alleviating mental fatigue and providing a sense of relaxation. According to Kaplan and Kaplan [23], a restorative environment should possess four key features: (1) fascination, the ability to attract involuntary effortless attention; (2) being away, the ability to trigger a psychological distance from daily routines; (3) compatibility, the ability to correspond to the individual’s expectations and the actual features of the environment; and (4) extent, the ability to provide an immersive experience within a coherent environment that facilitates sustained exploration. The component ‘extent’ comprises two elements: coherence, the ability to be perceived as structured and connected to one another; and scope, the ability to provide opportunities for exploration and involvement.

Research on perceived restorativeness has primarily compared natural and urban settings, with urban settings typically showing lower restorative values across cognitive and psychophysiological measures [24,25]. However, urban environments that incorporate natural elements—such as vegetation, water features, or naturalistic design—can also offer restorative benefits, including improved perceived restorativeness and cognitive functioning [26,27].

While urban environments often induce sensory overload and require sustained attention, UGSs can play a vital role in supporting well-being due to their accessibility and incorporation of natural features. There is growing evidence that urban settings such as museums [28], botanical gardens [29], urban landscapes [30], and historical sites [31] can also be perceived as restorative. Although much of the prior research has primarily focused on direct comparisons between purely natural and purely urban settings, this study extends existing work by including mixed nature-urban scenes, providing new insights into the restorative potential of urban settings with integrated natural elements.

### 1.3. Perceived naturalness

Perceived naturalness refers to the degree to which an environment is experienced as natural, based on human perception rather than ecological criteria [32,33]. Lamb and Purcell [34] distinguished this from ecological naturalness, which is defined by biological or ecological characteristics. While research on perceived naturalness remains relatively limited, existing studies have found a positive association between perceived naturalness and perceived restorativeness (e.g., Carrus et al. [29]). Similarly, Hipp et al. [35] found that the perceived greenness of a university campus positively correlated with perceived restorativeness, particularly for being away, fascination, and compatibility.

However, it is important to note that not all natural environments are inherently restorative. Some natural settings can evoke fear or discomfort, indicating that restorative benefits depend on how environments are interpreted and experienced [36]. It thus raises the question of whether the environment’s naturalness or its restorative qualities play a more significant role in promoting psychological and cognitive benefits. Shen et al. [37] argued that it is the subjective perception of naturalness—rather than the objective or typical representation of nature—that influences perceived restorativeness. Given this, the present study aims to explore not only the specific environmental features that influence perceptions of naturalness but also the relationship between perceived naturalness and perceived restorativeness. Establishing whether a positive correlation exists between these variables will contribute insights into the role of perceived naturalness in shaping restorative experiences, particularly in urban environments.

### 1.4. The impact of modalities on perceived restorativeness in natural environments

Research on restorative environments has primarily focused on visual natural stimuli, reflecting the visual system’s dominance in human perception [38–40]. Past researchers have been using different types of visual presentations, including 360-degree photos [41], 360-degree video [42], and visual stimuli presented through virtual reality (VR) [43]. Visual information primarily support the perception of “extent” and “compatibility”, thereby allowing individuals to assess the spatial scope of an environment and its alignment with their restorative needs [28].

However, visual input alone may not fully capture the restorative experience. Auditory perception, especially natural soundscapes, is increasingly recognized for its role in restoration. Auditory information may more directly influence “fascination” and “being away”, as natural soundscapes can capture attention and create psychological distance from stressors even when visual cues are ambiguous [44]. Studies have also shown that natural sounds promote greater psychological restoration than urban noise [45,46], and park settings with bird songs and minimal traffic noise are perceived as most restorative [47].

Recognizing the sensory richness of natural environments, recent research has begun to examine the combined effects of auditory and visual modalities on perceived restorativeness. Multisensory settings that integrate visual and natural auditory stimuli, such as birdsong and water sounds, are rated as more restorative than visual-only environments [48,49]. Audiovisual presentations may enhance restoration through cross-modal congruence, where matching visual and auditory features reinforce environmental authenticity and deepen immersion [50,51].

### 1.5. The current study

Despite the growing body of research on restorative environments, several important gaps remain. A key limitation lies in the narrow scope of environmental sampling. Most studies (e.g., [28,52,53]) rely on extreme contrasts between entirely natural and entirely urban environments. This approach overlooks the fact that most contemporary environments are mixed, blending both built and natural elements. As noted by Patuano [54] and Weber and Trojan [55], the lack of research on predominantly built and mixed urban settings highlights the need for a better understanding of the restorative potential of diverse urban configurations.

Furthermore, current restorativeness research lacks geographical diversity, as most data originate from temperate regions in Northern Europe, North America, and East Asia. These studies reflect specific ecological variables--such as distinct seasonality and temperate flora--that may not be representative of other climates and vegetative landscapes [56–58]. For instance, research in temperate China [59], South Korea [60], and the United Kingdom [61] has established robust patterns of restorative responses to manicured parks and seasonal greenery. However, tropical environments present fundamentally different ecological characteristics: year-round high temperatures and humidity, dense evergreen vegetation, distinct soundscapes dominated by insect and bird vocalizations, and different patterns of light penetration through canopy layers [62]. These differences raise critical questions about the generalizability of existing restorativeness frameworks. Do the same environmental features that promote restoration in temperate climates operate similarly in tropical contexts? Might the sensory intensity and biodiversity of tropical ecosystems alter the mechanisms through which restoration occurs?

To address this gap, the present study utilized environmental stimuli--nature, urban, and mixed urban-nature captured in Malaysia, a tropical landscapes in an equatorial climate, to examine perceived restorativeness across three modalities (visual-only, audio-only, and bimodal). We hypothesize that perceived restorativeness would vary across environmental scenes, such that nature and mixed urban-nature scenes will be perceived as more restorative than urban scenes (H1). Additionally, we also hypothesize that differences between environmental scenes would depend on modality (H2). Specifically, we hypothesize that the mixed urban-nature scenes will be perceived as similarly restorative to nature scenes within each modality condition (H2a). Moreover, it is also hypothesized that combined (audio-visual) presentations of nature scenes will have higher perceived restorativeness than those of single modality (i.e., visual and audio) (H2b). Finally, we also hypothesize that in these tropical climates, perceived naturalness would be positively associated with perceived restorativeness (H3).

## 2. Methodology

Participants were randomly allocated to one of three formats (video, visual, or audio) to rate a subset of 30 environmental scenes, out of a total of 120 stimuli. Each participant rated scenes in only one format. Participants were asked to rate a random subset of 30 environmental scenes rather than the full set, due to practical constraints. Rating the full set of 120 scenes would have significantly increased the time required to complete the study, which could have led to participant fatigue and decreased retention rates. By limiting the number of scenes to 30, we aimed to reduce the time burden on participants while maintaining sufficient diversity of environmental scene types. The environmental scenes were categorized into three types: nature mixed urban-nature, and urban.

### 2.1. Stimuli

Stimuli were collected from regions surrounding the Iskandar Puteri and Johor Bahru area within the Johor state in Malaysia. Environmental scenes were captured in both urban green space (UGS) and urban concrete space (UCS) within the area. Specifically, the stimuli encompassed scenes in Sireh Park, Hutan Bandar Mutiara Rini, Hutan Bandar Johor Bahru, Tanjung Piai, Puteri Harbour, Bukit Indah, and Eco Botanic. These stimuli highlight the lush, dense vegetation, and vibrant biodiversity characteristic of tropical environments, offering a contrast to the temperate landscapes often featured in similar studies conducted in Western contexts (e.g., [61,64]). The environmental scenes were systematically captured to represent a spectrum, ranging from predominantly natural settings (characterized by abundant amount of natural elements, with minimal man-made elements) to heavily urbanized settings (dominated by built elements with little to no natural features). This approach ensured a well-balanced representation of environment variation, highlighting the dynamic interplay between natural and urban features.

**Fig 1:**
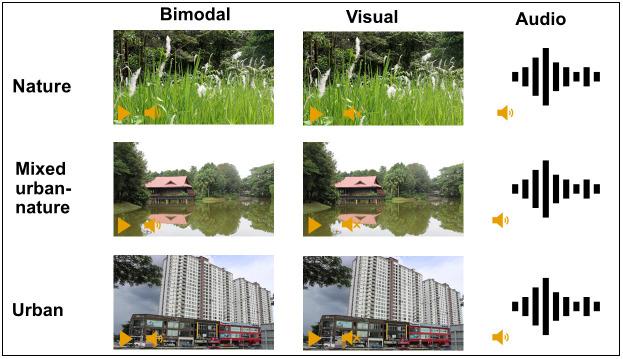
Examples of nine stimuli consisting of three types of environmental scenes and three modalities.

Video (along with audio) clips were shot using Canon DSLR camera with a constant setting, shutter of 1/50, aperture F16, and ISO 100. All multimedia were captured in full colours, and in horizontal mode. A total of 120 video stimuli on a continuum from nature to urban were collected and validated to be used in the study, each lasted for five seconds. The video stimuli were muted to produce visual-only clips, while audio clips were extracted from the video stimuli to produce audio-only clips (see Fig 1), resulting in a total of 360 stimuli.

### 2.2. Operational definition of a priori categorization of environmental scenes

The terms “natural environment” and “urban environment,” though widely understood by most, are ambiguous and subject to different interpretations. While we are aware of the risk of oversimplifying environmental scenes into dichotomies, we also acknowledge the profoundness of this distinction and its significance in understanding the impact of nature experiences on physical and psychological well-being (see Van den Berg [65] for review). In environmental psychology, ‘natural environment’ broadly refers to any outdoor or indoor place that is primarily covered by vegetation and other organic or inorganic natural components (such as rocks and water) [65]. Urban environment, on the other hand, often refers to any outdoor or indoor place where human-made built structures and facilities are predominantly present.

In this study, we categorized scenes with predominantly vegetation and natural components, and with no human-made elements, as nature scenes. Those with mostly human-made elements were classified as urban scenes. The mixed urban-nature category was included to represent environments where natural and human-made elements coexist. These spaces, often referred to as UGS, integrate vegetation, built infrastructure, and in some cases, blue elements such as sky and water bodies. Examples include urban parks where landscape greenery is interwoven with walking paths, benches, and architectural features, as well as areas where trees and shrubs border built structures like playgrounds, parking lots, or gazebos (see Fig 1, mixed urban-nature). After classifying the 120 stimuli, there were a total of 50 nature scenes, 50 urban scenes, and 20 mixed urban-nature scenes, in each modality. The mixed urban-nature category resulted in fewer classified stimuli because built features in tropical UGS are often partially obscured by dense vegetation or appear visually subtle. As a result, many otherwise suitable clips were excluded because they did not consistently meet our a priori criterion that both built and natural elements be clearly identifiable within the same scene.

### 2.3. Measures

The perceived restorativeness was measured using the short version of Korpela and Hartig’s [66] Perceived Restorativeness Scale. The original scale consists of 29 items and measures participants’ perception of five restorative components: fascination, being-away, coherence, scope, and compatibility. Berto [67] developed the Perceived Restorativeness Scales—Short Form (PR-SF) where it adopts a single item to measure each of the five components. The five items were rated on a 11-point Likert scale from 0 (*not at all*) to 10 (*completely*). Cronbach’s alpha reliability of the short version was.79 [67], indicating good reliability. Given that the PR-SF was primarily used to rate visual stimuli, we adapted a shortened 5-item Perceived Restorative Soundscape Scale—Short Form (PRS-SF) for the audio stimuli. This version was created by using the five items from Berto’s [67] PR-SF with language adapted from the PRS [68] (see Uebel et al. [47] for a similar approach). Table 1 shows the five items used in both scales. Although the PR-SF and PRS-SF differ in wording to reflect their respective modalities, item content was conceptually equivalent across both scales. Both scales operationalise the same five restorative components derived from ART [23], and cross-modality comparisons should therefore be interpreted as reflecting the same underlying construct.

**Table 1:** The five items used in PR-SF and PRS-SF.

Perceived Restorativeness Scale—Short Form (PR-SF) [67]	Perceived Restorative Soundscape Scale—Short Form (PRS-SF)
**Fascination:** This place is fascinating; it makes me wonder about things.	**Fascination**: The sounds in the recording have fascinating qualities; they make me wonder about things.
**Being-away**: This is a place which is away from everyday demands.	**Being-away**: The sounds in the recording give me a good break from my day-to-day routine.
**Coherence**: This is a place where the activities and the items are ordered and organized.	**Coherence**: The sounds in the recording seem to fit together quite well.
**Scope**: This is a place which is very large, with no restrictions to movements.	**Scope**: The sounds in the recording have a sense of depth and spaciousness.
**Compatibility**: In this place, it is easy to orient and move around so that I could do what I like.	**Compatibility**: The sounds in the recording relate to activities I like to do.

### 2.4. Procedure

The study was conducted online using Qualtrics. Participants were randomly assigned to one of three modality-specific blocks (audio-only, visual-only, or bimodal) using Qualtrics’ randomizer, such that each participant completed ratings in only one modality condition. Within the assigned block, stimulus presentation was implemented using Qualtrics’ Loop & Merge function. For each participant, the survey randomly selected 30 stimuli from the full pool of 120 and presented them in randomized order. Each stimuli played for five seconds, one at a time. Stimuli were presented as five-seconds clips to reduce participant burden in a high-volume rating task and helped maintain attention and response quality. Importantly, the study aimed to capture rapid, first-impression evaluations of perceived naturalness and restorativeness based on salient sensory cues. Prior restorative environment experiments have successfully used brief, standardised presentations to elicit such judgments—for example, presentation in which still images of natural settings were displayed for two seconds each [63].

Participants were instructed to carefully look/listen to each clip, and to imagine being in the place it depicted. Participants were not specifically instructed to use headphones (vs. speakers) during the audio and bimodal condition; therefore, playback device and listening context were not standardised. They were first required to rate each stimuli on perceived naturalness on a continuum from 0 (*not natural at all*) to 10 (*cannot be more natural*). Participants then evaluated the restorative value of each clip using five items from the PR-SF (for those assigned to the video and visual format) or the PRS-SF (for those assigned to the audio format).

### 2.5. Participants

Participants were recruited between February 2024 to April 2024. A total of 96 participants were recruited, but 9 sets of data were removed due to insufficient response effort, as the total response time was less than five minutes. The median time to completion was 24.23 minutes. Consequently, 87 participants (*M*_age_ = 25.62, *SD*_age_ = 7.74) were included in the analyses. 26 participants rated the video stimuli, while 29 participants rated the visual stimuli and 32 rated the audio stimuli. Sixty-two were female whereas twenty-five were males, and three participants prefer not to disclose their gender. The study was approved by the Ethical Committee of the University of Reading Malaysia, with ethical code UoRM REC 2024/02. Informed consent was collected from all participants via online consent form. A token of appreciation of RM 5 was provided to each participant upon completion of the study. Data were accessed for research purposes on 2 May 2024. The authors had access to identifiable information solely for participant reimbursement; all data were fully anonymized prior to analysis.

Because the primary analyses used a cross-classified linear mixed-effects model (participants and stimuli as random effects), an exact a priori power calculation was not available in G*Power. Statistical power for detecting the fixed environment * modality interaction was estimated using simulation-based methods via the “simr” package in R (version 4.4.1) and R studio (version 2024.09.0 + 375). Power was computed from 1,000 Monte Carlo simulations generated from the fitted model, with the interaction term tested using a likelihood ratio test (α = .05). This procedure indicated very high sensitivity for the interaction term (estimated power = 1.00, 95% CI [0.996, 1.00]). As this simulation was based on the effect sizes observed in the final dataset, the estimate should be interpreted as model-based post-hoc power.

As stimuli were randomly drawn without replacement within participants, each stimulus received ratings from an overlapping subset of participants. In the final dataset, each stimulus received between 3 and 14 ratings in the audio condition (*M* = 8.18, *SD* = 2.54), between 1 and 14 ratings in the bimodal condition (*M* = 6.50, *SD* = 2.22), and between 2 and 14 ratings in the visual condition (*M* = 7.25, *SD* = 2.50).

### 2.6. Analysis plan

All statistical analyses were conducted using R and R studio. Prior to hypothesis testing, a manipulation check was conducted to assess whether perceived naturalness differed significantly across the a priori categorization of environmental scenes. This was analysed using a linear mixed-effects model (LMM) with environment (nature, urban, mixed urban-nature) and modality (visual, audio, bimodal), and their interaction as fixed effects, and random intercepts for participants and stimuli.

The primary hypotheses were also tested using LMM, with perceived restorativeness as the dependent variable. Environment and modality were entered as fixed effects, along with their interactions. The model included random intercepts for participants and stimuli to account for variability across participants and stimulus items. The model was specified as: < lmerTest (Perceived restorativeness ~ Environment × Modality + (1 | Participant) + (1 | Stimulus)). All models were estimated using restricted maximum likelihood (REML) and converged without warnings. Random slopes for modality could not be estimated because participants were nested within modality conditions, and random slopes for environment resulted in singular fits, indicating insufficient variance to support their estimations.

Fixed effects were evaluated using Type III analysis of variance with F-tests. Denominator degrees of freedom and *p*-values for omnibus *F*-tests were estimated using Satterthwaite approximations. Where significant interaction effects were found, Post-hoc pairwise comparisons were conducted using estimated marginal means with Kenward-Roger degrees of freedom, with Bonferroni correction applied to control for multiple comparisons.

Specifically, simple effects of environment were examined within each modality (H2a), and simple effects of modality were examined within nature scenes (H2b). Then, H3 will be tested using a LMM with perceived restorativeness as the dependent variable and perceived naturalness as a predictor, including participants and stimuli as random intercepts. The model was specified as: < lmerTest(Perceived restorativeness ~ perceived naturalness + (1 | Participant) + (1 | Stimulus)).

## 3. Results

### 3.1. Manipulation checks

A manipulation check on perceived naturalness was conducted to verify the effectiveness of our a priori categorization of environmental scenes (nature, mixed urban-nature, urban). A linear mixed-effects analyses was fitted to perceive naturalness ratings, with environment (nature, urban, mixed urban-nature) and modality (visual, audio, bimodal) as fixed effects, and random intercepts for participants and stimuli. The results showed a significant main effect of environmental scene, *F*(2, 120.82) = 377.23, *p* < .001. There was also a significant interaction effect between environmental scene and modality, *F*(4, 2469.77) = 40.69, *p* < .001. Bonferroni-adjusted pairwise comparisons indicated that, across all modalities, nature and mixed urban–nature scenes were rated as higher in naturalness than urban scenes (all *p*s < .0001). Nature scenes were rated as more natural than mixed urban–nature scenes in the bimodal and visual conditions (both *p*s < .0001), but not in the audio condition (*p* = 1.00). The descriptive statistics and pairwise estimates are provided in Supplementary Materials S1 and S2 Tables, respectively.

Overall, the results are largely consistent with our a priori categorization of environmental scenes. Across modalities, nature and mixed urban-nature scenes were consistently rated as more natural than urban scenes. Nature scenes were also rated as more natural than mixed urban-nature scenes in the visual and bimodal conditions, but this difference did not emerge in the audio condition. This pattern suggests that auditory cues in mixed urban-nature scenes were perceived as comparably natural to those in fully natural scenes.

### 3.2. Main analyses

An overall perceived restorativeness score was computed by averaging ratings across the five restorative qualities (fascination, being away, coherence, scope, and compatibility). The means and standard deviations were presented in Table 2. The internal consistency of the perceived restorativeness composite score was acceptable to excellent across modalities, with Cronbach’s alpha values of.87 for the bimodal condition,.74 for the visual condition, and.95 for the audio condition. The ratings for each restorative qualities were included in the Supplementary Materials S3 Table.

**Table 2:** Descriptive statistics of perceived restorativeness.

Modalities	Environmental Scenes	n	Mean	Standard Deviation
Visual	Nature	360	5.84	1.98
	Urban	354	4.88	1.62
	Mixed urban-nature	156	6.49	1.66
	Total	870	5.56	1.88
Audio	Nature	372	5.90	2.46
	Urban	405	4.02	2.63
	Mixed urban-nature	183	5.64	2.71
	Total	960	5.05	2.73
Bimodal	Nature	316	6.53	2.14
	Urban	336	4.43	2.00
	Mixed urban-nature	128	6.61	1.95
	Total	780	5.64	2.30
Total	Nature	1048	6.07	2.23
	Urban	1095	4.42	2.18
	Mixed urban-nature	467	6.19	2.24
Total	Total	2610	5.40	2.36

#### 3.2.1. Hypothesis 1 (H1): Overall differences between environmental scenes.

A LMM was conducted to examine the effects of modalities and environmental scenes on perceived restorativeness. Intraclass correlation coefficients (ICCs) suggested that a substantial proportion of variance in perceived restorativeness was attributable to between-participant differences (ICC = .320, 95% CI [.279,.338]), with a smaller proportion attributable to between-stimulus differences (ICC = .038, 95% CI [.011,.036]).

The LMM analysis revealed a significant main effect of environment scene, *F*(2, 117.91) = 122.30, *p* < .001. To test H1, we examined Bonferroni-adjusted pairwise comparisons of model-estimated marginal means of environmental scenes averaged across modalities. Urban scenes were rated as significantly less restorative than both nature and mixed urban-nature scenes, both *p*s < .001. In contrast, mixed urban-nature scenes did not differ significantly from nature scenes (*p* = 1.000; Table 3). These findings partially support H1, indicating that nature scenes were perceived as more restorative than urban scenes, but not significantly more restorative than mixed urban-nature scenes.

**Table 3:** Pairwise contrasts between environmental scenes (averaged across modality) from the LMM.

Contrast	Contrast Estimates	SE	*df*	*t*	Adjusted *p*
Nature – Urban	1.68	0.11	118	14.71	<.001
Mixed urban-nature – Nature	0.12	0.15	114	0.82	1.000
Mixed urban-nature – Urban	1.80	0.15	112	12.10	<.001

Note. Contrasts are estimated marginal means averaged across modalities. As the environmental scene * modality interaction was significant, simple-effects contrast within each modality are reported in Table 4.

#### 3.2.2. Hypothesis 2 (H2): Environmental differences within each modality.

The same LMM model was used to examine the interaction effect between modality and environmental scene on perceived restorativeness. There was a significant interaction effect between modality and environmental scenes, *F*(4, 2473.64.21) = 14.01, *p* < .001. Post-hoc pairwise comparisons were conducted on the estimated marginal means derived from the mixed-effects model, revealing that the perceived restorativeness of environmental scenes depended on modality (see Table 4). For the visual condition, mixed urban-nature scenes were rated significantly more restorative than nature scenes (*p* = .001). Both nature and mixed urban-nature scenes were rated significantly more restorative than urban scenes (*p*s < .001). For the audio condition, nature and mixed urban-nature scenes did not differ significantly (*p* = .155), but both were rated as more restorative than urban scenes (*p*s < .001). For the bimodal condition, post-hoc comparisons yield similar results where nature and mixed urban-nature were rated higher in restorativeness than urban scenes (*p*s < .001), while no significant difference was found between nature and mixed urban-nature scenes (*p* = 1.000).

**Table 4:** Pairwise comparisons of perceived restorativeness between environmental scenes within each modality.

Modality	Contrast	Contrast Estimates	SE	*df*	*t*	Adjusted *p*
**Visual**	Nature – Urban	0.90	0.16	415	5.66	<.001
	Mixed urban-nature – Nature	0.72	0.21	384	3.53	.001
	Mixed urban-nature – Urban	1.62	0.21	381	7.94	<.001
**Audio**	Nature – Urban	2.02	0.16	372	13.11	<.001
	Mixed urban-nature – Nature	−0.38	0.20	324	−1.95	.155
	Mixed urban-nature – Urban	1.64	0.20	311	8.42	<.001
**Bimodal**	Nature – Urban	2.11	0.17	459	12.90	<.001
	Mixed urban-nature – Nature	0.02	0.22	476	0.11	1.000
	Mixed urban-nature – Urban	2.13	0.22	463	9.85	<.001

*Note*. Pairwise comparisons were conducted using estimated marginal means with Kenward-Roger degree of freedom and Bonferroni correction for multiple comparisons.

H2a predicted that mixed urban-nature scenes would be perceived as similarly restorative to nature scenes within each modality. This hypothesis is partially supported. In the bimodal and audio conditions, perceived restorativeness for nature and mixed urban-nature scenes did not differ significantly, indicating that participants perceive both types of environments as equally restorative. However, in the visual condition, participants rated mixed urban-nature scenes significantly higher in perceived restorativeness compared to nature scenes.

**Fig 2:**
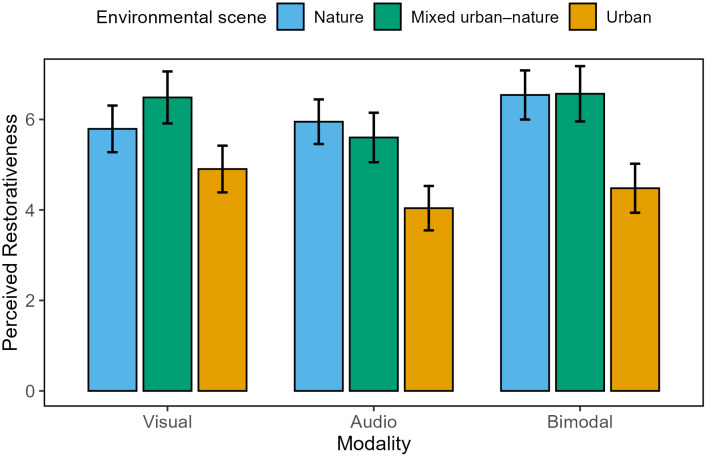
Estimated marginal means of perceived restorativeness across environmental scenes and modalities of experience derived from linear mixed-effects model. Note. Error bars represent 95% confidence intervals.

H2b examined whether, within nature scenes, bimodal presentation of stimuli would elicit higher perceived restorativeness than visual and audio stimuli. Pairwise comparisons of modality were performed within the nature condition, with Bonferroni correction applied. The results indicated no significant differences in perceived restorativeness between nature bimodal and nature visual presentations (contrast estimate = 0.75, *SE* = 0.36, *t*(100.6) = 2.06, *p* = .125), nature bimodal and nature audio presentations (contrast estimate = 0.55, *SE* = 0.36, *t*(101.3)= 1.55, *p* = .377), or nature visual and nature audio presentations (contrast estimate = −0.20, *SE* = 0.36, *t*(100.4)= −0.58, *p* = 1.000). Although the estimated marginal means suggested a trend favouring bimodal presentations (see Fig 2), these differences were not statistically significant. Thus H2b was not supported.

#### 3.2.3. Hypothesis 3 (H3): Association between perceived naturalness and perceived restorativeness.

To examine H3, we fitted an LMM predicting perceived restorativeness from standardized perceived naturalness, with random intercepts for participants and stimuli. There was a strong positive association between perceived naturalness and perceived restorativeness. Perceived naturalness was a significant predictor of perceived restorativeness, *F*(1, 1105.88) = 1401.30, *p* < .001. The fixed-effect estimate indicated that a one standard deviation increase in perceived naturalness was associated with a 1.34-point increase in perceived restorativeness (β = 1.34, SE = 0.04, *t* = 37.43, *p* < .001). Thus, H3 was suppor*t*ed.

## 4. Discussion

The present study aimed to evaluate the perceived restorativeness of common tropical landscapes within UGS in Malaysia across a range of environmental settings. In line with our hypothesis, nature scenes were rated as more restorative than urban scenes. This finding aligns with previous research on restorative environments, which has primarily focused on Western contexts (e.g., [28,61,63,64]). Our study extends these findings to Malaysia, a country with a tropical rainforest climate that differs markedly from the temperate climates commonly studied.

Unlike Western countries that experience four distinct seasons, Malaysia’s climate is consistently warm and humid, with seasonal variations driven mainly by rainfall rather than temperature [69]. These climatic conditions support dense year-round vegetation [70], high biodiversity [71], and distinctive soundscapes that are often dominated by singing insects [72] which may influence how sensory cues are interpreted as “natural” and restorative. Despite these differences, the restorative potential of natural environments observed in Western studies appears to extend to tropical climates as experienced in Malaysia. This suggests that the psychological benefits of exposure to natural settings may be broadly applicable across diverse climatic and cultural contexts. By using locally captured stimuli, this study contributes evidence from a tropical Southeast Asian context that is underrepresented in environmental psychology.

A key finding was the significant interaction between sensory modality and environment type. Nature and mixed urban-nature scenes were perceived as more restorative than urban scenes across all modalities. However, in the audio-only and bimodal conditions, mixed urban-nature scenes were rated as equally restorative as nature scenes. This is consistent with findings from Peron et al. [73] and with Ulrich’s [74] suggestion that aesthetically pleasing built environments—especially those featuring vegetation and water—can be restorative. Ulrich further suggested that individuals suffering from excessively low arousal or chronic boredom might benefit from being exposed to stimulating and vibrant urban environments.

Importantly, accessibility and quality of UGS are essential for optimizing their restorative potential. Features such as vegetation density, water elements, and spatial layout significantly enhance perceived restorativeness [75,76]. Berto [77] similarly argued that well-designed urban landscapes can substitute for natural environments in dense cities where pristine nature is less accessible. Mixed urban-nature scenes in our study demonstrated comparable restorativeness to nature scenes, reinforcing the importance of intentional design in supporting psychological well-being in urban settings [78,79].

Interestingly, in the visual modality alone, mixed urban-nature scenes were rated more restorative than nature scenes, while in the audio modality, there was no significant difference between them. Consistent with work showing that restorative power in UGS depend not only on the presence of vegetation but also on spatial configuration, mixed urban-nature scenes may have offered a comfortably structured green setting that supported higher perceived restorativeness [63]. In tropical environments, nature scenes are often characterized by dense, lush vegetation and reduced visibility depth, which can lower individual’s sense of coherence. In contrast, mixed urban-nature scenes often resembled UGS that provide a more structured layout with clearer edges and spatial organisation [80]. Li et al. [81] used eye-tracking data to demonstrate that increased ratios of hardscapes and buildings shorted average fixation duration, suggesting more efficient visual processing. Pure nature scenes, particularly dense vegetation, may present high visual complexity without sufficient structural organization, increasing cognitive load during visual processing [81]. Vosough et al. [82] found that the most restorative urban scenes featured trees on sidewalks and open front yards, suggesting that the integration of natural and built elements creates optimal visual structure. However, given that low-level visual properties (e.g., lighting, color saturation, and framing) were not controlled in this study, the observed visual-only advantage for mixed urban-nature scenes should be treated as preliminary and requires further validation.

The finding that bimodal presentation of nature scene offers no additional restorative benefit beyond unimodal conditions is consistent with several recent studies. Most notably, Shin et al. [83] found that adding birdsongs and natural scents to visual nature scenes presented through a virtual window did not provide restorative qualities beyond what the visual-only closed window condition offered. Similarly, Emfied et al [84] found that while participants rated the combined audiovisual nature presentation as most relaxing, this subjective preference did not translate into differential cognitive restoration effects. These results suggest that the “extent” component in ART appears to be achievable through a single sensory modality if that modality provides sufficient richness and coherence. A visually complex nature scene or a rich soundscape of layered natural sounds may each independently convey extent without requiring multisensory reinforcement.

We also found strong positive correlations between perceived naturalness and perceived restorativeness. This is consistent with Carrus et al. [29] and Shen et al. [37], who showed that environments perceived as more natural were rated as more restorative. According to Kaplan’s [17] ART, natural environments engage attention effortlessly, promoting mental recovery from fatigue. Ulrich et al.’s [18] Stress Reduction Theory also supports this, suggesting that natural features like greenery, water, and open space foster relaxation and reduce stress. While most prior research on perceived naturalness has focused on temperate cities, our study is among the first to systematically examine this relationship in a tropical context. Related work in subtropical Asia, such as Chang et al. [85], found that greater bird species richness enhanced both perceived naturalness and perceived restorativeness in Taiwan. Hwang et al. [86] observed that Singaporeans preferred moderately wild UGS and found that elements like flowers, fauna, and boardwalks increased preferences for wilder landscapes. Future studies could explore how people from Western cultures respond to tropical environments, offering cross-cultural insights into naturalness and perceived restorativeness perceptions in UGS.

The current study advances previous work by integrating both visual and auditory stimuli from consistent environmental settings, rather than combining disparate stimuli. For example, while Jahncke et al. [87] demonstrated that auditory stimuli (e.g., nature sounds, office noise, and silence) affected the perceived restorativeness of visual environments (nature or office), their stimuli were not always from matching contexts. By ensuring that both visual and auditory inputs came from the same environment, our design enhances ecological validity and provides a more realistic simulation of real-world experiences, rather than combining stimuli from separate settings, as seen in previous research (e.g., [48,87]).

Several limitations of the present study should be noted. The study was conducted online, which meant that audio playback conditions were not fully standardised (e.g., headphone versus speaker use), potentially introducing variability in auditory perception. Additionally, the five-seconds stimulus clips likely capture initial appraisals of perceived restorativeness rather than the full time-course of restoration, as restorative processes may deepen with longer or more immersive exposure. On the other hand, although the PR-SF and PRS-SF were designed to operationalise the same restorative components, the modality-specific wording of items may introduce measurement variance that limits the interpretability of direct cross-modality comparisons. The number of stimuli across environmental categories was also not evenly distributed, with the mixed urban-nature category containing fewer stimuli than the other categories, which may reduce the precision of estimates for that category. Additionally, the a priori classifications may not fully align with participants’ subjective perceptions; for instance, nature and mixed urban-nature scenes in the audio condition did not differ significantly in perceived naturalness, underscoring the challenge of categorising tropical environments using discrete scene labels. Future research could benefit from complementing categorical labels with tools such as the Natural Environment Scoring Tool (NEST; [88]). Regarding sample composition, the sample was predominantly female (71.62%), which may limit generalisability to the broader population.

Building on the present findings, future research should examine how restorative responses observed through virtual exposure translate to real-world environments, particularly in tropical contexts where climatic conditions may shape environmental experience in distinct ways. Field-based investigations in UGSs would allow for testing whether the benefits identified under controlled conditions generalise to more immersive, everyday encounters with nature. In addition, combining subjective ratings with physiological or behavioural measures (e.g., salivary cortisol, eye-tracking, or time spent in a space) could yield a more comprehensive understanding of multimodal restorative experiences.

## 5. Conclusion

This study is among the first to comprehensively evaluate perceived restorativeness of nature, urban, and mixed urban-nature scenes captured from UGS in a tropical context. By providing perceived restorativeness ratings for a diverse set of 360 stimuli, it contributes a valuable dataset on tropical cities, an area underrepresented in restorative environment research.

We show that mixed urban-nature scenes, which combine natural and built elements, were perceived as equally restorative as nature-only scenes. This suggests that built environments with substantial vegetation can offer restorative benefits comparable to predominantly natural settings. Additionally, bimodal presentation of nature scenes did not yield higher perceived restorativeness than visual or audio presentation, indicating that multisensory input may not confer additive benefits when unimodal natural cues are already salient. Beyond expanding the geographical scope of restorative environment research, this study provides a validated framework for assessing the restorativeness of diverse UGSs. The dataset and findings serve as a foundation for future studies, including investigations into whether individuals from non-tropical regions perceive tropical stimuli similarly to local residents. Importantly, the results offer guidance for urban planning and design strategies aimed at optimizing the restorative potential of UGSs, especially in densely populated tropical cities.

## Supporting information

S1 Table Descriptive statistics of perceived naturalness. (DOCX)

S2 Table Pairwise comparisons of perceived naturalness between environmental scenes within each modality. (DOCX)

S3 Table Means and standard deviations of the five components of perceived restorativeness. (DOCX)

## References

[1] United Nations. 2018 Revision of World Urbanization Prospects. United Nations Department of Economic and Social Affairs. [Internet]. [cited 2025 Oct 9]. Available from: https://www.un.org/en/desa/2018-revision-world-urbanization-prospects

[2] Chen J, Chen S, Landry PF, Davis DS. How dynamics of urbanization affect physical and mental health in urban China. China Q. 2014;220:988–1011. doi: 10.1017/s0305741014001465

[3] Lecic-Tosevski D. Is urban living good for mental health? Curr Opin Psychiatry. 2019;32(3):204–9. doi: 10.1097/YCO.0000000000000489 30695002

[4] Berman MG, Jonides J, Kaplan S. The cognitive benefits of interacting with nature. Psychol Sci. 2008;19(12):1207–12. doi: 10.1111/j.1467-9280.2008.02225.x 19121124

[5] Berman MG, Kross E, Krpan KM, Askren MK, Burson A, Deldin PJ, et al. Interacting with nature improves cognition and affect for individuals with depression. J Affect Disord. 2012;140(3):300–5. doi: 10.1016/j.jad.2012.03.012 22464936 PMC3393816

[6] Bratman GN, Daily GC, Levy BJ, Gross JJ. The benefits of nature experience: improved affect and cognition. Landsc Urban Plan. 2015;138:41–50. doi: 10.1016/j.landurbplan.2015.02.005

[7] Han K-T. The effect of nature and physical activity on emotions and attention while engaging in green exercise. Urban For Urban Green. 2017;24:5–13. doi: 10.1016/j.ufug.2017.03.012

[8] Liu L, Qu H, Ma Y, Wang K, Qu H. Restorative benefits of urban green space: physiological, psychological restoration and eye movement analysis. J Environ Manage. 2022;301:113930. doi: 10.1016/j.jenvman.2021.113930 34731949

[9] Neill C, Gerard J, Arbuthnott KD. Nature contact and mood benefits: contact duration and mood type. J Posit Psychol. 2019;14(6):756–67. doi: 10.1080/17439760.2018.1557242

[10] Song S, Tu R, Lu Y, Yin S, Lin H, Xiao Y. Restorative effects from green exposure: a systematic review and meta-analysis of randomized control trials. Int J Environ Res Public Health. 2022;19(21):14506. doi: 10.3390/ijerph192114506 36361386 PMC9658851

[11] Wood E, Harsant A, Dallimer M, Cronin de Chavez A, McEachan RRC, Hassall C. Not all green space is created equal: biodiversity predicts psychological restorative benefits from urban green space. Front Psychol. 2018;9:2320. doi: 10.3389/fpsyg.2018.02320 30538653 PMC6277587

[12] Annerstedt M, Jönsson P, Wallergård M, Johansson G, Karlson B, Grahn P, et al. Inducing physiological stress recovery with sounds of nature in a virtual reality forest--results from a pilot study. Physiol Behav. 2013;118:240–50. doi: 10.1016/j.physbeh.2013.05.023 23688947

[13] Lim PY, Dillon D, Chew PKH. A guide to nature immersion: psychological and physiological benefits. Int J Environ Res Public Health. 2020;17(16):5989. doi: 10.3390/ijerph17165989 32824731 PMC7459647

[14] World Health Organization (WHO). Urban Green Spaces: A Brief for Action. Geneva: World Health Organization; 2017 [cited 2025 Oct 9]. Available from: https://www.who.int/europe/publications/i/item/9789289052498

[15] Corkery L. Urban green space. Routledge eBooks. Routledge; 2019. pp. 294–304. doi: 10.4324/9781351211543-32

[16] Kumuduni Y, Weerakoon KGPK. Defining urban green spaces in the Colombo district across multiple uses. JRES. 2024;21(2). doi: 10.31357/jres.v21i2.7543

[17] Kaplan S. The restorative benefits of nature: toward an integrative framework. J Environ Psychol. 1995;15(3):169–82. doi: 10.1016/0272-4944(95)90001-2

[18] Ulrich RS, Simons RF, Losito BD, Fiorito E, Miles MA, Zelson MF. Stress recovery during exposure to natural and urban environments. J Environ Psychol. 1991;11(3):201–30. doi: 10.1016/S0272-4944(05)80184-7

[19] Burtan D, Joyce K, Burn JF, Handy TC, Ho S, Leonards U. The nature effect in motion: visual exposure to environmental scenes impacts cognitive load and human gait kinematics. R Soc Open Sci. 2021;8(1):201100. doi: 10.1098/rsos.201100 33614067 PMC7890511

[20] Ulrich RS. Visual landscapes and psychological well-being. Landsc Res. 1979;4(1):17–23. doi: 10.1080/01426397908705892

[21] McMahan EA, Estes D. The effect of contact with natural environments on positive and negative affect: a meta-analysis. J Posit Psychol. 2015;10(6):507–19. doi: 10.1080/17439760.2014.994224

[22] Kondo MC, Jacoby SF, South EC. Does spending time outdoors reduce stress? A review of real-time stress response to outdoor environments. Health Place. 2018;51:136–50. doi: 10.1016/j.healthplace.2018.03.001 29604546

[23] Kaplan R, Kaplan S. The experience of nature: A psychological perspective. Cambridge University Press; 1989.

[24] Franěk M, Šefara D, Petružálek J, Cabal J, Myška K. Differences in eye movements while viewing images with various levels of restorativeness. J Environ Psychol. 2018;57:10–6. doi: 10.1016/j.jenvp.2018.05.001

[25] Grassini S, Revonsuo A, Castellotti S, Petrizzo I, Benedetti V, Koivisto M. Processing of natural scenery is associated with lower attentional and cognitive load compared with urban ones. J Environ Psychol. 2019;62:1–11. doi: 10.1016/j.jenvp.2019.01.007

[26] Tenngart Ivarsson C, Hagerhall CM. The perceived restorativeness of gardens – Assessing the restorativeness of a mixed built and natural scene type. Urban For Urban Green. 2008;7(2):107–18. doi: 10.1016/j.ufug.2008.01.001

[27] San Juan C, Subiza-Pérez M, Vozmediano L. Restoration and the city: the role of public urban squares. Front Psychol. 2017;8:2093. doi: 10.3389/fpsyg.2017.02093 29270139 PMC5725966

[28] Stragà M, Miani C, Mäntylä T, De Bruin WB, Mottica M, Del Missier F. Into the wild or into the library? Perceived restorativeness of natural and built environments. J Environ Psychol. 2023;91:102131. doi: 10.1016/j.jenvp.2023.102131

[29] Carrus G, Lafortezza R, Colangelo G, Dentamaro I, Scopelliti M, Sanesi G. Relations between naturalness and perceived restorativeness of different urban green spaces. PsyEcol: Bilingual J Environ Psychol. 2013;4(3):227–44. doi: 10.1174/217119713807749869

[30] Karmanov D, Hamel R. Assessing the restorative potential of contemporary urban environment(s): Beyond the nature versus urban dichotomy. Landsc Urban Plan. 2008;86(2):115–25. doi: 10.1016/j.landurbplan.2008.01.004

[31] Wang S, Zhang L, Xu Y, Jiang Y, Gao Y, Leng Y. Determining how historical sites create value. Int Rev Spat Plan Sustain Dev. 2023;11(2):150–67. doi: 10.14246/irspsd.11.2_150

[32] Ode A, Fry G, Tveit MS, Messager P, Miller D. Indicators of perceived naturalness as drivers of landscape preference. J Environ Manage. 2009;90(1):375–83. doi: 10.1016/j.jenvman.2007.10.013 18280633

[33] Tveit M, Ode Å, Fry G. Key concepts in a framework for analysing visual landscape character. Landsc Res. 2006;31(3):229–55. doi: 10.1080/01426390600783269

[34] Lamb RJ, Purcell AT. Perception of naturalness in landscape and its relationship to vegetation structure. Landsc Urban Plan. 1990;19(4):333–52. doi: 10.1016/0169-2046(90)90041-y

[35] Hipp JA, Gulwadi GB, Alves S, Sequeira S. The relationship between perceived greenness and perceived restorativeness of university campuses and student-reported quality of life. Environ Behav. 2016;48(10):1292–308. doi: 10.1177/0013916515598200

[36] Van den Berg AE, Ter Heijne M. Fear versus fascination: an exploration of emotional responses to natural threats. J Environ Psychol. 2005;25(3):261–72. doi: 10.1016/j.jenvp.2005.08.004

[37] Shen H, He X, He J, Li D, Liang M, Xie X. Back to the village: assessing the effects of naturalness, landscape types, and landscape elements on the restorative potential of rural landscapes. Land. 2024;13(7):910. doi: 10.3390/land13070910

[38] Celikors E, Wells NM. Are low-level visual features of scenes associated with perceived restorative qualities? J Environ Psychol. 2022;81:101800. doi: 10.1016/j.jenvp.2022.101800

[39] Franco LS, Shanahan DF, Fuller RA. A review of the benefits of nature experiences: more than meets the eye. Int J Environ Res Public Health. 2017;14(8):864. doi: 10.3390/ijerph14080864 28763021 PMC5580568

[40] Li Z, Ba M, Kang J. Physiological indicators and subjective restorativeness with audio-visual interactions in urban soundscapes. Sustain Cities Soc. 2021;75:103360. doi: 10.1016/j.scs.2021.103360

[41] Glennon J, Barton H. Virtual nature, mindfulness, and the potential for altruism. Routledge eBooks. Routledge; 2018. pp. 22–33. doi: 10.4324/9781315160962-3

[42] Litleskare S, Calogiuri G. Camera stabilization in 360° videos and its impact on cyber sickness, environmental perceptions, and psychophysiological responses to a simulated nature walk: a single-blinded randomized trial. Front Psychol. 2019;10:2436. doi: 10.3389/fpsyg.2019.02436 31736832 PMC6839361

[43] Lakhani A, Martin K, Gray L, Mallison J, Grimbeek P, Hollins I, et al. What Is the impact of engaging with natural environments delivered via virtual reality on the psycho-emotional health of people with spinal cord injury receiving rehabilitation in hospital? Findings from a pilot randomized controlled trial. Arch Phys Med Rehabil. 2020;101(9):1532–40. doi: 10.1016/j.apmr.2020.05.013 32502564

[44] Boggs JB. The roles of biophilic attitudes and auditory stimuli within attention restoration theory. Las Vegas (NV): University of Nevada, Las Vegas; 2018.

[45] Abbott LC, Taff D, Newman P, Benfield JA, Mowen AJ. The influence of natural sounds on attention restoration. J Park Recreat Adm. 2016;34(3). doi: 10.18666/jpra-2016-v34-i3-6893

[46] Krzywicka P, Byrka K. Restorative qualities of and preference for natural and urban soundscapes. Front Psychol. 2017;8:1705. doi: 10.3389/fpsyg.2017.01705 29046653 PMC5632731

[47] Uebel K, Marselle M, Dean AJ, Rhodes JR, Bonn A. Urban green space soundscapes and their perceived restorativeness. People Nat. 2021;3(3):756–69. doi: 10.1002/pan3.10215

[48] Deng L, Luo H, Ma J, Huang Z, Sun L-X, Jiang M-Y, et al. Effects of integration between visual stimuli and auditory stimuli on restorative potential and aesthetic preference in urban green spaces. Urban For Urban Green. 2020;53:126702. doi: 10.1016/j.ufug.2020.126702

[49] Zhao J, Xu W, Li Y. Effects of auditory-visual combinations on perceived restorative potential of urban green space. Appl Acoust. 2018;141:169–77. doi: 10.1016/j.apacoust.2018.07.001

[50] Xu W, Xu S, Shi R, Chen Z, Lin Y, Chen J. Exploring the impact of university green spaces on students’ perceived restoration and emotional states through audio-visual perception. Ecol Inform. 2024;82:102766. doi: 10.1016/j.ecoinf.2024.102766

[51] Zhang H, Zhu Z, Zhang D-W. Designing sustainable urban green spaces: audio-visual interaction for psychological restoration. Sustainability. 2025;17(19):8906. doi: 10.3390/su17198906

[52] Neale C, Lopez S, Roe J. Psychological restoration and the effect of people in nature and urban scenes: a laboratory experiment. Sustainability. 2021;13(11):6464. doi: 10.3390/su13116464

[53] Schaupp J, Hediger K, Wunderli J-M, Schäffer B, Tobias S, Kolecka N, et al. Psychophysiological effects of walking in forests and urban built environments with disparate road traffic noise exposure: study protocol of a randomized controlled trial. BMC Psychol. 2024;12(1):250. doi: 10.1186/s40359-024-01720-x 38711162 PMC11073983

[54] Patuano A. Biophobia and urban restorativeness. Sustainability. 2020;12(10):4312. doi: 10.3390/su12104312

[55] Weber AM, Trojan J. The restorative value of the urban environment: a systematic review of the existing literature. Environ Health Insights. 2018;12. doi: 10.1177/1178630218812805 30505146 PMC6256310

[56] Rockstroh C, Blum J, Hardt V, Göritz AS. Design and evaluation of a virtual restorative walk with room-scale virtual reality and impossible spaces. Front Virtual Real. 2020;1. doi: 10.3389/frvir.2020.598282

[57] Payne SR. Are perceived soundscapes within urban parks restorative. J Acoust Soc Am. 2008;123(5_Supplement):3809. doi: 10.1121/1.2935525

[58] Gidlow CJ, Jones MV, Hurst G, Masterson D, Clark-Carter D, Tarvainen MP. Where to put your best foot forward: psycho-physiological responses to walking in natural and urban environments. J Environ Psychol. 2015;45:22–9. doi: 10.1016/j.jenvp.2015.11.003

[59] Lu X, Xu J, Lange E, Cao J. Which factors enhance the perceived restorativeness of streetscapes: sound, vision, or their combined effects? Insights from four street types in Nanjing, China. Land. 2025;14(4):757. doi: 10.3390/land14040757

[60] Tae J, Jeong D, Chon J. How can apartment-complex landscaping space improve residents’ psychological well-being?: The case of the capital region in South Korea. Int J Environ Res Public Health. 2022;19(16):10231. doi: 10.3390/ijerph191610231 36011865 PMC9408058

[61] Lai KY, Sarkar C, Sun Z, Scott I. Are greenspace attributes associated with perceived restorativeness? A comparative study of urban cemeteries and parks in Edinburgh, Scotland. Urban For Urban Green. 2020;53:126720. doi: 10.1016/j.ufug.2020.126720

[62] Yap T, Dillon D, Chew PKH. The impact of nature imagery and mystery on attention restoration. Multidiscip Sci J. 2022;5(4):478–99. doi: 10.3390/j5040033

[63] Van den Berg AE, Jorgensen A, Wilson ER. Evaluating restoration in urban green spaces: does setting type make a difference? Landsc Urban Plan. 2014;127:173–81. doi: 10.1016/j.landurbplan.2014.04.012

[64] Twedt E, Rainey RM, Proffitt DR. Beyond nature: the roles of visual appeal and individual differences in perceived restorative potential. J Environ Psychol. 2019;65:101322. doi: 10.1016/j.jenvp.2019.101322

[65] van den Berg AE. The natural-built distinction in environmental preference and restoration: bottom-up and top-down explanations. Nebraska Symposium on Motivation. Springer International Publishing; 2021. pp. 31–60. doi: 10.1007/978-3-030-69020-5_3

[66] Korpela K, Hartig T. Restorative qualities of favorite places. J Environ Psychol. 1996;16(3):221–33. doi: 10.1006/jevp.1996.0018

[67] Berto R. Exposure to restorative environments helps restore attentional capacity. J Environ Psychol. 2005;25(3):249–59. doi: 10.1016/j.jenvp.2005.07.001

[68] Payne SR. The production of a perceived restorativeness soundscape scale. Appl Acoust. 2013;74(2):255–63. doi: 10.1016/j.apacoust.2011.11.005

[69] Alhoot MA, Tong WT, Low WY, Sekaran SD. Climate Change and Health: The Malaysia Scenario. Advances in Asian Human-Environmental Research. Springer International Publishing; 2016. pp. 243–68. doi: 10.1007/978-3-319-23684-1_15

[70] Lewis SL. Tropical forests and the changing earth system. Philos Trans R Soc Lond B Biol Sci. 2006;361(1465):195–210. doi: 10.1098/rstb.2005.1711 16553317 PMC1626535

[71] Cooper DLM, Lewis SL, Sullivan MJP, Prado PI, Ter Steege H, Barbier N, et al. Consistent patterns of common species across tropical tree communities. Nature. 2024;625(7996):728–34. doi: 10.1038/s41586-023-06820-z 38200314 PMC10808064

[72] Riede K, Balakrishnan R. Acoustic monitoring for tropical insect conservation. Philos Trans R Soc Lond B Biol Sci. 2025;380(1928):20240046. doi: 10.1098/rstb.2024.0046 40501134 PMC12180399

[73] Peron E, Berto R, Purcell T. Restorativeness, preference and the perceived naturalness of places. Medio Ambient Comport Hum. 2002;3(1):19–34.

[74] Ulrich RS. Aesthetic and affective response to natural environment. Springer eBooks. 1983. pp. 85–125. doi: 10.1007/978-1-4613-3539-9_4

[75] Nisbet EK, Zelenski JM. Underestimating nearby nature. Psychol Sci. 2011;22(9):1101–6. doi: 10.1177/0956797611418527 21828351

[76] Zuo W, Cheng B, Feng X, Zhuang X. Relationship between urban green space and mental health in older adults: mediating role of relative deprivation, physical activity, and social trust. Front Public Health. 2024;12:1442560. doi: 10.3389/fpubh.2024.1442560 39267636 PMC11390600

[77] Berto R. The role of nature in coping with psycho-physiological stress: a literature review on restorativeness. Behav Sci (Basel). 2014;4(4):394–409. doi: 10.3390/bs4040394 25431444 PMC4287696

[78] Li Y, Zhang J, Jiang B, Li H, Zhao B. Do all types of restorative environments in the urban park provide the same level of benefits for young adults? A field experiment in Nanjing, China. Forests. 2023;14(7):1400. doi: 10.3390/f14071400

[79] Yeh C-W, Hung S-H, Chang C-Y. The influence of natural environments on creativity. Front Psychiatry. 2022;13:895213. doi: 10.3389/fpsyt.2022.895213 35966494 PMC9363772

[80] Felappi JF, Sommer JH, Falkenberg T, Terlau W, Kötter T. Urban park qualities driving visitors mental well-being and wildlife conservation in a Neotropical megacity. Sci Rep. 2024;14(1):4856. doi: 10.1038/s41598-024-55357-2 38418539 PMC10902329

[81] Li C, Yuan Y, Sun C, Sun M. The perceived restorative quality of viewing various types of urban and rural scenes: based on psychological and physiological responses. Sustainability. 2022;14(7):3799. doi: 10.3390/su14073799

[82] Ahmadınazhad Vosough S, Bozkurt M. Evaluating the restorative potential of different green strategies in streets. Adnan Menderes Üniversitesi Ziraat Fakültesi Dergisi. 2024;21(2):145–53. doi: 10.25308/aduziraat.1447614

[83] Shin S, Browning MHEM, Dzhambov AM. Window access to nature restores: a virtual reality experiment with greenspace views, sounds, and smells. Ecopsychology. 2022;14(4):253–65. doi: 10.1089/eco.2021.0032

[84] Emfield AG, Neider MB. Evaluating visual and auditory contributions to the cognitive restoration effect. Front Psychol. 2014;5:548. doi: 10.3389/fpsyg.2014.00548 24926279 PMC4046122

[85] Chang J, Wu C-C, Chang C-Y. Landscape naturalness and restoring benefit: a connection through bird diversity. Urban Ecosyst. 2023;27(1):41–50. doi: 10.1007/s11252-023-01425-w

[86] Hwang YH, Yue ZEJ, Ling SK, Tan HHV. It’s ok to be wilder: preference for natural growth in urban green spaces in a tropical city. Urban For Urban Green. 2019;38:165–76. doi: 10.1016/j.ufug.2018.12.005

[87] Jahncke H, Eriksson K, Naula S. The effects of auditive and visual settings on perceived restoration likelihood. Noise Health. 2015;17(74):1–10. doi: 10.4103/1463-1741.149559 25599752 PMC4918645

[88] Gidlow C, van Kempen E, Smith G, Triguero-Mas M, Kruize H, Gražulevičienė R, et al. Development of the natural environment scoring tool (NEST). Urban For Urban Green. 2018;29:322–33. doi: 10.1016/j.ufug.2017.12.007

