# Supplementary material for: Sensing nature in the city: The role of sight and sound in restorative tropical urban green spaces
Source: PLoS One. 2026 Jun 15;21(6):e0351647. doi: 10.1371/journal.pone.0351647 (PMC13268155; doi:10.1371/journal.pone.0351647)
Supplement: S1 Table — (DOCX) [file pone.0351647.s001.docx]

**S1 Table**. Descriptive statistics of perceived naturalness.

| Modality | Environmental Scene | n | Mean | Standard Deviation |
| --- | --- | --- | --- | --- |
| Visual | Nature | 360 | 8.34 | 1.96 |
|  | Urban | 353 | 3.78 | 2.58 |
|  | Mixed urban-nature | 157 | 7.04 | 2.22 |
| Audio | Nature | 372 | 6.94 | 2.61 |
|  | Urban | 398 | 4.65 | 2.92 |
|  | Mixed urban-nature | 183 | 6.81 | 2.73 |
| Bimodal | Nature | 316 | 8.26 | 1.99 |
|  | Urban | 336 | 3.30 | 2.16 |
|  | Mixed urban-nature | 128 | 6.92 | 2.38 |
