# Supplementary material for: Sensing nature in the city: The role of sight and sound in restorative tropical urban green spaces
Source: PLoS One. 2026 Jun 15;21(6):e0351647. doi: 10.1371/journal.pone.0351647 (PMC13268155; doi:10.1371/journal.pone.0351647)
Supplement: S2 Table — (DOCX) [file pone.0351647.s002.docx]

**S2 Table**. Pairwise comparisons of perceived naturalness between environmental scenes within each modality.

| Modality | Contrast | Estimates | SE | *df* | *t* | *p*-value |
| --- | --- | --- | --- | --- | --- | --- |
| Visual | Nature – Urban | 4.60 | 0.20 | 372 | 23.07 | < .001 |
|  | Nature – Mixed urban-nature | 1.22 | 0.26 | 342 | 4.73 | < .001 |
|  | Mixed urban-nature - Urban | 3.38 | 0.26 | 340 | 13.19 | <.001 |
| Audio | Nature – Urban | 2.29 | 0.20 | 338 | 11.78 | < .001 |
|  | Nature – Mixed urban-nature | 0.24 | 0.25 | 294 | 0.97 | 1.000 |
|  | Mixed urban-nature - Urban | 2.05 | 0.25 | 285 | 8.34 | < .001 |
| Bimodal | Nature – Urban | 4.95 | 0.20 | 409 | 24.26 | <.001 |
|  | Nature – Mixed urban-nature | 1.27 | 0.27 | 423 | 4.67 | <.001 |
|  | Mixed urban-nature – Urban | 3.69 | 0.27 | 412 | 13.63 | <.001 |
