# Supplementary material for: Sensing nature in the city: The role of sight and sound in restorative tropical urban green spaces
Source: PLoS One. 2026 Jun 15;21(6):e0351647. doi: 10.1371/journal.pone.0351647 (PMC13268155; doi:10.1371/journal.pone.0351647)
Supplement: S3 Table — (DOCX) [file pone.0351647.s003.docx]

**S3 Table**. Means and standard deviations of the five components of perceived restorativeness

|  | Nature | | Mixed urban-nature | | Urban | |
| --- | --- | --- | --- | --- | --- | --- |
|  | *M* | *SD* | *M* | *SD* | *M* | *SD* |
| **Fascination**  Bimodal  Visual  Audio | 6.86  5.93  5.91 | 2.76  2.80  2.67 | 6.52  6.24  5.97 | 2.43  2.62  2.90 | 3.82  4.06  4.24 | 2.37  2.67  2.85 |
| **Being-away**  Bimodal  Visual  Audio | 6.96  6.79  5.61 | 2.53  2.57  2.92 | 6.70  6.29  5.33 | 2.63  2.40  3.08 | 3.37  3.59  3.77 | 2.37  2.61  3.02 |
| **Coherence**  Bimodal  Visual  Audio | 5.30  4.49  6.12 | 2.86  2.68  2.63 | 6.28  6.19  5.86 | 2.25  2.13  2.86 | 5.76  6.44  4.46 | 2.74  2.28  3.03 |
| **Scope**  Bimodal  Visual  Audio | 6.91  6.29  6.11 | 2.44  2.60  2.60 | 6.73  6.75  5.74 | 2.36  2.11  2.86 | 4.57  5.11  4.17 | 2.54  2.17  2.96 |
| **Compatibility**  Bimodal  Visual  Audio | 6.63  5.69  5.56 | 2.66  2.64  2.95 | 6.81  6.86  5.39 | 2.22  2.00  3.23 | 4.61  5.18  3.80 | 2.60  2.31  3.01 |
