# Supplementary material for: Sensing nature in the city: The role of sight and sound in restorative tropical urban green spaces
Source: PLoS One. 2026 Jun 15;21(6):e0351647. doi: 10.1371/journal.pone.0351647 (PMC13268155; doi:10.1371/journal.pone.0351647)
Supplement: Attachment — Submitted filename: Response to Reviewers.docx [file pone.0351647.s004.docx]

Manuscript PONE-D-25-54884

Dear Reviewers,

Thank you for the careful and constructive feedback. A major revision in the current manuscript concerns our statistical approach and analysis pipeline. We agree that the original between-subjects ANOVA conducted on stimulus-averaged scores was not well aligned with the study design because participants rated only a subset of stimuli. Accordingly, we have re-analysed the data using linear mixed-effects modelling (LMM) in R, treating trial-level ratings (participants * stimulus) as the unit of analysis and modelling participants and stimuli as random effects. Environmental scenes, modalities, and their interaction were entered as fixed effects. To ensure consistency and reproducibility, we transitioned all analyses from SPSS to R, including descriptive statistics, manipulation checks, reliability analyses, and the power analysis.

These changes resulted in several updates to the Results. First, manipulation checks were re-analysed using an LMM predicting perceived naturalness. The overall pattern remained consistent with the a priori categorisation, except that nature and mixed urban-nature scenes did not differ significantly in perceived naturalness in the audio modality. Second, we estimated the intraclass correlation coefficients directly from the fitted mixed-effects model, instead of aggregating ratings at the stimulus level. Third, internal consistency of the perceived restorativeness composite score increased substantially when computed at the trial level. Finally, although the interaction between environment and modality remained significant, post-hoc tests no longer supported the hypothesis that bimodal presentations of nature scenes yield higher perceived restorativeness than single-modality presentations. We have updated the manuscript accordingly and provide point-by-point responses below, indicating where revisions were made.

Response to Reviewer 1

| Comments | Responses |
| --- | --- |
| **Use of stimuli as the primary unit of analysis** The manuscript treats stimuli, rather than participants, as the main unit of analysis. This raises the issue of pseudoreplication, because stimuli are not independent observations: multiple videos come from the same location, share visual/auditory properties, and were rated by overlapping sets of participants. While stimuli-level analyses are not inherently inappropriate, this decision must be justified—particularly in a high-variance, naturalistic stimulus set. Recommendations: Justify the use of stimuli as the unit of analysis and acknowledge potential pseudoreplication. If possible, include a supplementary analysis with a linear mixed-effects model treating participants and stimuli as random effects. Even a simplified version (e.g., only for the visual or bimodal modality) would significantly reinforce the statistical credibility of the work. Expand the Limitations section to discuss this issue and its implications. | Thank you for raising this important concern. We agree that our original stimulus-aggregated ANOVA approach risked pseudoreplication because observations were not independent (ratings were cross-classified by both participants and stimuli). In response, we have revised the analytical approach substantially.  Specifically, we re-analysed the full dataset using linear mixed-effects modelling, treating trial-level ratings (participant * stimulus) as the unit of analysis and modelling participants and stimuli as random intercepts. This approach directly accounts for the non-independence in the data by partitioning variance attributable to individuals and stimulus items, thereby addressing the pseudoreplication issue inherent in stimulus-level aggregation.  As a result of this revision, the manuscript no longer treats stimuli as the primary unit of analysis. We have updated the Methods (Line 347 – 365) to describe the mixed-effects specification, revised the Results (Line 370– 475) to report mixed-model fixed effects. |
| **Imbalanced number of stimuli across categories** The three environmental categories are unevenly represented: 50 nature 50 urban 20 mixed This large imbalance may distort ANOVA variance estimates and limit interpretability of interaction effects. The authors should provide more detail on how and why the mixed category contains fewer scenes. Was it inherently difficult to identify mixed scenes in tropical settings? Were selection criteria stricter? Recommendations: Offer a clearer explanation for the imbalance. Consider a balanced subsampling analysis (e.g., 20 scenes per category), even as supplementary material. This would show whether key findings (e.g., the nature vs. mixed effects) remain robust. | Thank you for noting the imbalance in the number of stimuli across environmental categories. The main analysis has now been re-analysed using linear mixed-effects modelling. This approach is more tolerant of unequal cell sizes than ANOVA and does not rely on balancing stimulus counts across conditions in the same way as an ANOVA design. Nevertheless, we acknowledge that underrepresentation of the mixed category may reduce precision for estimates involving mixed urban-nature scenes, and we have noted this as a limitation (Line 572 – 575).  We also clarified the practical reasons for the smaller mixed urban-nature set. Mixed urban-nature scenes were defined a priori as clips containing both prominent natural elements and built features. During stimulus selection, many potential scenes in tropical urban green spaces were visually dominated by dense vegetation; built elements were often present but subtle and thus did not consistently meet our operational definition of “mixed”. As a result, fewer stimuli satisfied the criteria without becoming either predominantly natural or predominantly urban. We have added this clarification to the subsection 2.2 (Line 271 – 276) |
| **Low internal consistency in the visual-only condition (α = .42)** The Cronbach’s alpha reported for the visual-only condition is very low. This suggests instability in participants’ responses and calls into question the interpretability of the interaction effects involving the visual modality. Recommendations: Explore whether one or more items (e.g., Scope) reduced reliability. Consider reporting McDonald’s omega. Discuss why reliability was low only for visual stimuli and how this affects interpretation. | Thank you for highlighting the low internal consistency previously reported for the visual-only condition (α = .42).  In the revised manuscript, we recomputed reliability using the trial-level data (i.e., item responses nested within Participant * Stimulus), consistent with the revised analytic approach. Under this approach, internal consistency for the visual-only condition increased substantially to an acceptable level (α = .74). The bimodal and audio conditions showed good to excellent reliability (α = .87 and α = .95, respectively).  We have updated the Results accordingly and corrected interpretations that relied on the earlier reliability estimate (Line 392 - 395). |
| **Interpretation of mixed urban-nature scenes** A key finding is that participants rated mixed scenes as equally natural as nature scenes, particularly in the visual-only modality. This challenges the operational distinction between categories. Figure 1 indicates that the “mixed” scenes contain substantial vegetation, which may explain the perceptual overlap. Recommendations: Clarify the criteria used to classify scenes as “mixed”. Discuss the possibility that mixed scenes were sufficiently dense in vegetation to be perceived as “natural”. Expand the theoretical discussion on the complexity of categorizing tropical environments, where vegetation can dominate even mixed settings. | Thank you for highlighting the potential ambiguity in our mixed urban–nature classification. In the revised manipulation checks (analysed using LMM), nature and mixed urban–nature scenes were distinguishable in the visual-only and bimodal conditions (nature > mixed), but not in the audio-only condition.  We expanded the Discussion (Line 576 - 582) to acknowledge that categorising tropical environments can be inherently challenging because vegetation is prevalent and may dominate sensory impressions.  To strengthen transparency and replicability in future research, we also recommend complementing categorical labels with objective environmental indices (e.g., Natural Environment Scoring Tool), which quantify features such as vegetation density and ecological richness, and integrating these measures with subjective ratings of perceived naturalness and restorativeness. |
| **Stimulus duration (5 seconds)** The choice of 5-second clips should be more clearly justified. Restorative perception can be slow to emerge, and many studies use longer durations. Recommendations: Provide a stronger rationale supported by literature. Mention this as a limitation given the cognitive/emotional processes involved. | Thank you for pointing that out. We clarified the rationale for using 5-s clips (Line 300 - 306) and added a limitation (Line 590 - 592). Clips were kept brief to standardize exposure and reduce fatigue given the number of trials, and because prior work indicates that environmental evaluations can be formed from brief exposures. We also acknowledge that longer exposures may be required to capture slower-emerging restorative processes and recommend this direction for future research. |
| **Post-hoc tests (Fisher’s LSD)** The use of Fisher’s LSD should be justified, as it increases the risk of Type I errors, particularly in factorial designs with multiple comparisons. Recommendations: Either justify the use of LSD (e.g., planned comparisons) or Re-run post-hoc tests using Tukey HSD or Sidak and report whether conclusions remain unchanged. | We agree that Fisher’s LSD is comparatively liberal and can increase the risk of Type I error in factorial designs with multiple comparisons. In the revised manuscript, we have therefore replaced Fisher’s LSD with post-hoc comparisons based on estimated marginal means from the linear mixed-effects model, applying Bonferroni correction to control the family-wise error rate for the sets of planned pairwise contrasts. |
| **Cultural and climatic context** The study’s geographic context is a major strength, and the manuscript could highlight this more explicitly. Perception of nature and restorative qualities likely varies depending on cultural familiarity with tropical vegetation, biodiversity, or humid climate. Recommendations: Expand the section discussing cultural and climatic influences on perception. Emphasize how this work contributes to increasing geographic diversity in environmental psychology research. | We expanded the Introduction (Line 186 – 200) and Discussion (Line 486 – 489; 493 – 495; 518 - 520) to more explicitly situate the study in tropical context and to explain why climate differences may shape perceptions of naturalness and restorativeness. We also clarified how the study addresses the geographic concentration of restorative environments research by providing evidence from a tropical Southeast Asian setting. |
| **Clarity and structure** The manuscript is clearly written, though some conceptual explanations (ART vs. SRT) could be streamlined. | Thank you for this suggestion. We have now streamlined Section 1.1 and 1.3 to improve clarity and reduce redundancy. |
| **Figures** In Figure 1, modality icons partially obscure the scenes; consider reducing icon size or opacity. Ensure high-resolution versions of all figures. | Thank you for this suggestion. We improved Figure 1 by reducing the size of the modality icons. We also ensured that all figures are provided in high-resolution formats consistent with the journal’s requirements. |
| **Procedures** Clarify how many participants rated each stimulus. Provide more detail on the exclusion criteria (e.g., <5 min completion time) and typical completion time. Specify whether participants used headphones or speakers, as this influences auditory perception. | Participants were randomly assigned to one modality and rated 30 random stimuli. We now report the resulting range and average number of ratings per stimulus within each modality in the Methods (338 - 342).  To clarify, participants with <5 mins completion time were excluded from analysis. We’ve now added the median time of completion in Line 318 - 319 (24.23 minutes).  We clarified in the Methods (Line 308 – 310) that headphone use was not mandated and that audio playback conditions were not standardised; we also noted this as a limitation (Line 593 - 595) because listening context may influence auditory perception. |
| Statistics Report effect sizes consistently (e.g., ηp²). Provide descriptive statistics for naturalness across categories in supplementary material. Provide ICC confidence intervals for transparency. | Thank you for this suggestion.  Descriptive statistics for naturalness across categories have been included in Table S1 in supplementary material.  Rather than reporting ηp², which can be difficult to interpret in linear mixed-effects models with unbalanced or complex random structures, we have provided full parameter estimates (*β*) with 95% Confidence Intervals and *t*-values for all fixed effects. These values offer a direct measure of the magnitude and direction of the relationships, which is more aligned with the predictive nature of mixed-effects modeling.  To improve transparency regarding the nested structure of the data, we now report intraclass correlation coefficients (ICCs) with 95% confidence intervals derived from the variance components of the cross-classified mixed-effects model. |

Respond to Reviewer 2

| From a conceptual standpoint, the introduction provides a broad overview of theories and constructs (ART, SRT, naturalness, multisensory integration), but it would benefit from a more focused and theoretically cohesive narrative. At present, the motivation for the study feels somewhat descriptive rather than driven by a clear theoretical gap. It would be helpful to more explicitly articulate why tropical environments might differ from those studied in temperate climates, and how multisensory cues theoretically relate to restorative processes. More importantly, the hypotheses would be stronger if they emerged more directly from this theoretical framing rather than simply reflecting expected differences between scene categories. | Thank you for this constructive feedback. In the revised manuscript, we now explicitly articulate why tropical environments may present different sensory and interpretive conditions than the temperate contexts that dominate the literature (Line 186 - 200). We also clearly link multisensory cues to restorative processes through ART mechanisms in the Introduction (Line 159 – 161; 164 – 166; 174 – 176). |
| --- | --- |
| My main concern is that the statistical approach is strongly inappropriate for the study design. Your analysis treats stimuli as the unit of analysis (N = 360), averaging participant ratings for each stimulus and then running a between-subjects 3X3 ANOVA. The main issue is that this is not a 3x3 ANOVA. Participants did not rate all stimuli; thus, their effects cannot be controlled. Your design probably requires a mixed-effects model given the nested nature of your data. | We sincerely thank the reviewer for this critical insight. We agree that the initial ANOVA approach, which utilized stimuli as the unit of analysis, did not sufficiently account for the nested structure of our data.  In accordance with your recommendation, we have completely re-analyzed the data using the linear mixed-effects model. This approach is appropriate for our design as it allows us to treat both participants and stimuli as random effects. By doing so, we account for the non-independence of observations and the inherent variability across different participants and stimuli. |
| Cronbach’s α for the perceived restorativeness scores for visual group condition is 0.42, which is far below acceptable psychometric standards. | Thank you for raising the concern. In our initial draft, alpha was calculated on aggregated data which did not account for the nested structure of our design.  In the revised manuscript (Line 392 – 395), we recomputed reliability using trial-level data (treating item responses as nested within participant and stimulus), which is consistent with our move to a linear mixed-effects framework. Under this more appropriate analytic approach, the internal consistency for the visual-only condition increased to an acceptable level (α = .74). The audio and audio-visual (bimodal) conditions also demonstrated strong reliability (α = .95 and α = .87, respectively). |
| Another issue is related to the stimuli categorization. Although the manuscript provides a priori definitions for nature, mixed, and urban scenes, the manipulation check reveals that participants did not perceive a difference between nature and mixed scenes across any modality. This raises conceptual ambiguity: if the two categories are perceptually indistinguishable, then the theoretical distinction underlying your hypotheses becomes unclear. Effectively, the mixed urban–nature stimuli were too similar to the purely natural ones. In most cases, the ‘urban’ component in the mixed scenes was minimal or barely perceptible, which likely contributed to the absence of clear perceptual differences between the two categories. | Thank you for raising this important point. We agree that the distinction between nature and mixed urban–nature scenes is central to interpreting the hypotheses.  In the revised manipulation checks (analysed using LMM), nature and mixed urban–nature scenes were distinguishable in the visual-only and bimodal conditions (nature > mixed), but not in the audio-only condition.  We also acknowledge that in tropical UGS, dense vegetation may visually dominate scenes such that built components can be perceptually less salient.  We expanded the Discussion (Line 576 – 582) to emphasise that this pattern does not invalidate the mixed urban-nature category, but rather highlights an ecologically meaningful feature of tropical UGS: mixed urban-nature environments may nevertheless be experienced as functionally “natural” depending on the salience of sensory cues. |
| The very high correlation between perceived naturalness and restorativeness in the audio group (r = .92) raises the possibility that naturalness ratings and restorative qualities overlap semantically, and that the two measures capture similar constructs. I suggest the authors to acknowledge this limitation in the discussion. | Thank you for this insightful observation regarding the potential semantic overlap between perceived naturalness and restorativeness. We agree that the initial correlation of *r* = .92 was remarkably high, suggesting a possible lack of discriminant validity in the auditory condition.  In our original analysis, we examined the association between perceived naturalness and perceived restorativeness using Pearson’s correlation based on average stimulus ratings, with stimuli as the unit of analysis. While this approach provided an initial indication of the relationship, it did not account for the nested structure of the data, in which multiple participants rated multiple stimuli.  To address this, we have now tested the association using a linear mixed-effects model (LMM: Perceived restorativeness ~ Naturalness + (1\|Participant_ID) + (1\|Stimulus)).  The regression coefficient (β = 1.34) (Line 468 – 475) suggests that while naturalness is a powerful predictor of perceived restoration, the two scales remain psychometrically distinct. |
| The interpretation of the interaction between modality and scene type would benefit from a more cautious approach. The result that mixed scenes were perceived as more restorative than purely natural scenes in the visual-only condition is certainly interesting, but the explanation offered (i.e., absence of urban noise enhances visual restorativeness) remains somewhat speculative. There are several other plausible reasons why this pattern might have emerged. For example, the mixed scenes may have simply been more visually coherent or compositionally balanced, or they might have contained greater scenic complexity that viewers found engaging. It is also possible that aesthetic preferences, unrelated to the presence of urban elements, played a role, or that uncontrolled visual features such as lighting, color saturation, or framing influenced these judgments. I encourage you to acknowledge these alternative explanations and to present a more tempered interpretation of this finding. | Thank you for this helpful suggestion. We agree that our initial interpretation was somewhat speculative. We have revised the Discussion (Line 515 – 532) to adopt a more cautious interpretation of the finding that mixed urban-nature scenes were rated as more restorative than nature scenes in the visual-only condition. Specifically, we now frame this pattern as potentially reflecting differences in spatial configuration and visual legibility (e.g., clearer edges, greater spatial organisation, and higher visibility depth) in mixed urban-nature scenes. We also acknowledge that we did not experimentally control low-level visual properties (e.g., lighting, colour saturation) which may have contributed to the observed differences. |
| The rationale for limiting clips to 5 seconds could be expanded. | Thank you for the suggestion. We agree that the duration of stimuli is a critical factor in restoration research. We have expanded the rationale in Line 300 – 306 to better articulate why 5 seconds was chosen. |
| Please report instructions regarding the use of headphones for audio stimuli. | We clarified in the Methods (Line 308 – 310) that headphone use was not mandated and that audio playback conditions were not standardised; we also noted this as a limitation (Line 593 – 595) because listening context may influence auditory perception. |
| Gender imbalance (62 F, 22 M) could be addressed as a limitation. | We agree that the gender distribution in our sample is a limitation that may affect the generalizability of our findings. The imbalance was a result of convenience sampling method adopted for this study. We have included this as a limitation (Line 599 - 603) acknowledging the future research should aim for a more balanced recruitment strategy. |
| Provide information on randomization procedure in Qualtrics. | We have updated the manuscript (Line 294 – 299) to provide a more detailed description of the randomization procedure. |
| Table 2: ICC for fascination in visual-only is extremely low (0.39); discuss implications. | Thank you for pointing that out. Following the transition to LMM, we have recomputed the ICCs based on variance components (the ratio of random effect variance to total variance) (Line 402 – 406). This approach provides a much clearer understanding of the data's reliability, where both between-participant and between-stimulus ICC were computed. |
