# Supplementary material for: Sensing nature in the city: The role of sight and sound in restorative tropical urban green spaces
Source: PLoS One. 2026 Jun 15;21(6):e0351647. doi: 10.1371/journal.pone.0351647 (PMC13268155; doi:10.1371/journal.pone.0351647)
Supplement: Attachment — Submitted filename: Response to Reviewers Letter.docx [file pone.0351647.s005.docx]

Manuscript PONE-D-25-54884R2

We thank the Reviewers for the thorough and constructive feedback on our revised manuscript. We are pleased that the Reviewer found the shift to trial-level cross-classified linear mixed-effects model to be a substantial improvement and that the manuscript is considered close to be publishable. Below, we address each comment in detail.

Response to Reviewer 1

| Comments | Responses |
| --- | --- |
| At present, I could not verify that a full reproducibility package is publicly available via a stable repository link/DOI (or as complete Supporting Information). Before acceptance, please provide: (i) the trial-level dataset (participant ID anonymized, stimulus ID, modality, environment category, naturalness, the 5 restorativeness items, and any exclusions), (ii) a codebook/metadata file, (iii) the analysis scripts (R code) required to reproduce all results/figures/tables, and (iv) a stimulus ID mapping file linking each stimulus ID to the corresponding audio/visual/bimodal files and the a priori scene categorization. If media files cannot be shared publicly (e.g., licensing/privacy), please state exactly what cannot be shared and why, and provide an ethics-approved access route consistent with PLOS policy. | Thank you for highlighting this important requirement. We have now deposited a complete reproducibility package on the Open Science Framework (OSF). The repository is organised into two components to separate stimulus materials from study-specific data and analysis files.  Main project: Tropical Urban Green Space Restorativeness Project  DOI: <https://doi.org/10.17605/OSF.IO/4CBYQ>  Component 1 - Tropical Environmental Stimuli Database (TESD)   - Stimulus files: The complete set of 120 environmental stimuli (audio, visual, and bimodal formats) used in the study. - Stimulus mapping file (Stimulus_Mapping.csv): links each stimulus ID to its corresponding media filenames and a priori environment categorisation.   Component 2 – Sensing Nature In The City: The Role of Sight and Sound in Tropical Urban Green Spaces   - Trial-level dataset (long_clean_dataset_with_PRS.xlsx): Contains anonymised participant IDs, stimulus IDs, modality condition, environment category, naturalness ratings, the five restorative items, and the composite perceived restorativeness score. Excluded participants (n = 9) are not included in the dataset; exclusion criteria are described in the manuscript (Section 2.5). - Codebook (Codebook.md): Describes each variable, including measurement scales, factor levels, and data processing steps. - Analysis scripts (LMM_Latest.Rmd): Contains all R code required to reproduce the reported results, figures, and tables. |
| Please report the LMM specification transparently (exact model formulae; df method such as Kenward–Roger/Satterthwaite; brief diagnostics/convergence; whether random slopes were explored and, if not, why). | Thank you for this suggestion. We have revised the Analysis Plan (Section 2.6) to report the LMM specification more transparently. The revised text now includes:   1. Exact model formula: The exact model formula is stated in Line 359 –360. 2. Degrees of freedom method: Omnibus F-tests used Satterthwaite approximations for denominator degrees of freedom and *p*-values. Post-hoc pairwise comparisons used Kenward-Roger degrees of freedom with Bonferroni correction. (Line 366 – 367). 3. Convergence and diagnostics: All models converged without warnings. Inspection of scaled residuals indicated no substantial departures from model assumptions. (Line 360 –361) 4. Random slopes: Random slopes for modality could not be estimated because participants were nested within modality conditions (each participant rated stimuli in only one modality). Random slopes for environment were explored but resulted in singular fits, indicating insufficient variance to support their estimation. (Line 361 – 364). |
| Because the audio condition uses adapted soundscape wording for the restorativeness items, please strengthen justification for cross-modality comparability and/or temper interpretation accordingly (a brief sensitivity check would be welcome). | Thank you for flagging this up. We have strengthened the justification for cross-modality comparability in the Measures section (Line 290 – 294). Specifically, we have added text clarifying that although the PR-SF and PRS-SF differ in surface-level wording to reflect their respective modalities, both scales operationalise the same five restorative components drawn from ART. This adaptation follows the same rationale as Uebel et al. (2021), whom we cited in the original manuscript.  Given this conceptual alignment (grounded in shared theoretical structure and a precedent in the literature) and the item-level correspondence across scales, we consider the measures sufficiently comparable for the purposes of the present analyses. Nonetheless, as a matter of methodological transparency, we acknowledge in the Discussion (Line 586 – 590) that modality-specific wording may introduce measurement variance that limits the interpretability of direct cross-modality comparisons. |
| Please clarify how the updated power analysis was performed in the LMM framework (ideally simulation-based), ensuring it matches the inferential approach. | Thank you for this suggestion. We have revised the power analysis description in Section 2.5 (Line 336 – 339) to clarify that simulation-based methods were used. Specifically, power was estimated using the “simr” package in R, which generates Monte Carlo simulations from the fitted cross-classified LMM. The Environment × Modality interaction was tested using a likelihood ratio test, matching the inferential approach used in the main analyses. |
| Consider consolidating limitations in a clearly labelled paragraph (online setting; unstandardized audio playback; brief exposure duration; sample composition; geographic generalizability). | Thank you for this suggestion. We have consolidated the limitations into the final paragraphs of the Discussion (Lines 581 – 609), addressing methodological considerations (online setting, unstandardised audio playback, brief exposure duration, modality-specific measures) as well as limitations relating to stimuli and sample characteristics. We have also reframed the future research directions positively, emphasising how subsequent studies can build on the present findings. |
