# Supplementary material for: Sensing nature in the city: The role of sight and sound in restorative tropical urban green spaces
Source: PLoS One. 2026 Jun 15;21(6):e0351647. doi: 10.1371/journal.pone.0351647 (PMC13268155; doi:10.1371/journal.pone.0351647)
Supplement: Peer review history

# Peer review: Decision Letter 0

**DOI:** 10.1371/journal.pone.0351647.r001

**Authors:** Annalisa Theodorou

26 Dec 2025

PONE-D-25-54884

Sensing nature in the city: The role of sight and sound in restorative tropical urban green spaces

PLOS One

Dear Dr. Hoo Ju Yun,

Thank you for submitting your manuscript to PLOS ONE. After careful consideration, we feel that it has merit but does not fully meet PLOS ONE’s publication criteria as it currently stands. Therefore, we invite you to submit a revised version of the manuscript that addresses the points raised during the review process.

==============================

I received the opinions of two reviewers, and I independently assessed the manuscript myself.  I agree with both reviewers in recognizing the value of examining multisensory restorative perception, particularly in tropical urban green spaces; however, substantial revisions are required to strengthen the work.

In revising the manuscript, please make sure to address every point raised by the reviewers. Including: their concern regarding treating stimuli as the unit of analysis; the low internal consistency of the visual-only condition (Cronbach’s α = .42); the fact that mixed scenes were often perceived as indistinguishable from purely natural scenes, particularly in the visual modality; a more cautious and nuanced interpretation of the interaction effects between modality and scene type; the use of short (5-second) stimulus clips should be more thoroughly justified and discussed as a limitation; the tropical and cultural context of the study is a significant strength that could be more clearly and coherently integrated into the theoretical framework.

==============================

Please submit your revised manuscript by Feb 09 2026 11:59PM. If you will need more time than this to complete your revisions, please reply to this message or contact the journal office at plosone@plos.org. When you're ready to submit your revision, log on to https://www.editorialmanager.com/pone/ and select the 'Submissions Needing Revision' folder to locate your manuscript file.

Please include the following items when submitting your revised manuscript:

- A letter that responds to each point raised by the academic editor and reviewer(s). You should upload this letter as a separate file labeled 'Response to Reviewers'.
- A marked-up copy of your manuscript that highlights changes made to the original version. You should upload this as a separate file labeled 'Revised Manuscript with Track Changes'.
- An unmarked version of your revised paper without tracked changes. You should upload this as a separate file labeled 'Manuscript'.

If you would like to make changes to your financial disclosure, please include your updated statement in your cover letter. Guidelines for resubmitting your figure files are available below the reviewer comments at the end of this letter.

If applicable, we recommend that you deposit your laboratory protocols in protocols.io to enhance the reproducibility of your results. Protocols.io assigns your protocol its own identifier (DOI) so that it can be cited independently in the future. For instructions see: https://journals.plos.org/plosone/s/submission-guidelines#loc-laboratory-protocols. Additionally, PLOS ONE offers an option for publishing peer-reviewed Lab Protocol articles, which describe protocols hosted on protocols.io. Read more information on sharing protocols at https://plos.org/protocols?utm_medium=editorial-email&utm_source=authorletters&utm_campaign=protocols.

We look forward to receiving your revised manuscript.

Kind regards,

Annalisa Theodorou

Academic Editor

PLOS One

Journal Requirements:

When submitting your revision, we need you to address these additional requirements.

1. Please ensure that your manuscript meets PLOS ONE's style requirements, including those for file naming. The PLOS ONE style templates can be found at

https://journals.plos.org/plosone/s/file?id=wjVg/PLOSOne_formatting_sample_main_body.pdf and

https://journals.plos.org/plosone/s/file?id=ba62/PLOSOne_formatting_sample_title_authors_affiliations.pdf

2. Your ethics statement should only appear in the Methods section of your manuscript. If your ethics statement is written in any section besides the Methods, please delete it from any other section.

3. We are unable to open your Supporting Information file [Data.sav]. Please kindly revise as necessary and re-upload.

4. If the reviewer comments include a recommendation to cite specific previously published works, please review and evaluate these publications to determine whether they are relevant and should be cited. There is no requirement to cite these works unless the editor has indicated otherwise.

[Note: HTML markup is below. Please do not edit.]

Reviewers' comments:

Reviewer's Responses to Questions

**Comments to the Author**

1. Is the manuscript technically sound, and do the data support the conclusions?

The manuscript must describe a technically sound piece of scientific research with data that supports the conclusions. Experiments must have been conducted rigorously, with appropriate controls, replication, and sample sizes. The conclusions must be drawn appropriately based on the data presented.

Reviewer #1: Partly

Reviewer #2: Partly

**********

2. Has the statistical analysis been performed appropriately and rigorously?

Reviewer #1: Yes

Reviewer #2: No

**********

3. Have the authors made all data underlying the findings in their manuscript fully available?

The PLOS Data policy requires authors to make all data underlying the findings described in their manuscript fully available without restriction, with rare exception (please refer to the Data Availability Statement in the manuscript PDF file). The data should be provided as part of the manuscript or its supporting information, or deposited to a public repository. For example, in addition to summary statistics, the data points behind means, medians and variance measures should be available. If there are restrictions on publicly sharing data—e.g. participant privacy or use of data from a third party—those must be specified.

Reviewer #1: Yes

Reviewer #2: Yes

**********

4. Is the manuscript presented in an intelligible fashion and written in standard English?

PLOS ONE does not copyedit accepted manuscripts, so the language in submitted articles must be clear, correct, and unambiguous. Any typographical or grammatical errors should be corrected at revision, so please note any specific errors here.

Reviewer #1: Yes

Reviewer #2: Yes

**********

5. Review Comments to the Author

Please use the space provided to explain your answers to the questions above. You may also include additional comments for the author, including concerns about dual publication, research ethics, or publication ethics. (Please upload your review as an attachment if it exceeds 20,000 characters)

Reviewer #1: Thank you for the opportunity to review this manuscript. The study addresses an important and underexplored topic: the perceptual and restorative qualities of tropical urban green spaces (UGS), and how they vary across sensory modalities. The work is timely, well-motivated, and methodologically ambitious, relying on a large and diverse set of stimuli. The manuscript is clearly written and contributes meaningfully to environmental psychology and urban planning, particularly in non-Western contexts. However, several methodological and analytical issues require clarification or strengthening. My recommendation is Major Revision. Below I provide detailed comments structured to support the improvement of the manuscript.

1. Overall Assessment

The manuscript is scientifically relevant and well framed. It extends prior work—mostly based on temperate or Western settings—by focusing on a tropical context. The authors collected 360 stimuli and used a 3 × 3 factorial design to evaluate perceived restorativeness across three environmental categories and three sensory modalities. The results are interesting and generally well aligned with the Attention Restoration Theory (ART) and Stress Reduction Theory (SRT).

However, several methodological issues require attention before the work can be considered technically robust enough for publication. These issues relate mainly to the definition and balance of the stimuli categories, unit of analysis, reliability in the visual modality, and interpretation of the mixed environments. Addressing these concerns will greatly increase the rigor, clarity, and impact of the manuscript.

2. Major Comments

2.1. Use of stimuli as the primary unit of analysis

The manuscript treats stimuli, rather than participants, as the main unit of analysis. This raises the issue of pseudoreplication, because stimuli are not independent observations: multiple videos come from the same location, share visual/auditory properties, and were rated by overlapping sets of participants. While stimuli-level analyses are not inherently inappropriate, this decision must be justified—particularly in a high-variance, naturalistic stimulus set.

Recommendations:

Justify the use of stimuli as the unit of analysis and acknowledge potential pseudoreplication.

If possible, include a supplementary analysis with a linear mixed-effects model treating participants and stimuli as random effects. Even a simplified version (e.g., only for the visual or bimodal modality) would significantly reinforce the statistical credibility of the work.

Expand the Limitations section to discuss this issue and its implications.

2.2. Imbalanced number of stimuli across categories

The three environmental categories are unevenly represented:

50 nature

50 urban

20 mixed

This large imbalance may distort ANOVA variance estimates and limit interpretability of interaction effects. The authors should provide more detail on how and why the mixed category contains fewer scenes. Was it inherently difficult to identify mixed scenes in tropical settings? Were selection criteria stricter?

Recommendations:

Offer a clearer explanation for the imbalance.

Consider a balanced subsampling analysis (e.g., 20 scenes per category), even as supplementary material.

This would show whether key findings (e.g., the nature vs. mixed effects) remain robust.

2.3. Low internal consistency in the visual-only condition (α = .42)

The Cronbach’s alpha reported for the visual-only condition is very low. This suggests instability in participants’ responses and calls into question the interpretability of the interaction effects involving the visual modality.

Recommendations:

Explore whether one or more items (e.g., Scope) reduced reliability.

Consider reporting McDonald’s omega.

Discuss why reliability was low only for visual stimuli and how this affects interpretation.

2.4. Interpretation of mixed urban-nature scenes

A key finding is that participants rated mixed scenes as equally natural as nature scenes, particularly in the visual-only modality. This challenges the operational distinction between categories. Figure 1 indicates that the “mixed” scenes contain substantial vegetation, which may explain the perceptual overlap.

Recommendations:

Clarify the criteria used to classify scenes as “mixed”.

Discuss the possibility that mixed scenes were sufficiently dense in vegetation to be perceived as “natural”.

Expand the theoretical discussion on the complexity of categorizing tropical environments, where vegetation can dominate even mixed settings.

2.5. Stimulus duration (5 seconds)

The choice of 5-second clips should be more clearly justified. Restorative perception can be slow to emerge, and many studies use longer durations.

Recommendations:

Provide a stronger rationale supported by literature.

Mention this as a limitation given the cognitive/emotional processes involved.

2.6. Post-hoc tests (Fisher’s LSD)

The use of Fisher’s LSD should be justified, as it increases the risk of Type I errors, particularly in factorial designs with multiple comparisons.

Recommendations:

Either justify the use of LSD (e.g., planned comparisons)

or

Re-run post-hoc tests using Tukey HSD or Sidak and report whether conclusions remain unchanged.

2.7. Cultural and climatic context

The study’s geographic context is a major strength, and the manuscript could highlight this more explicitly. Perception of nature and restorative qualities likely varies depending on cultural familiarity with tropical vegetation, biodiversity, or humid climate.

Recommendations:

Expand the section discussing cultural and climatic influences on perception.

Emphasize how this work contributes to increasing geographic diversity in environmental psychology research.

3. Minor Comments

Clarity and structure

The manuscript is clearly written, though some conceptual explanations (ART vs. SRT) could be streamlined.

Figures

In Figure 1, modality icons partially obscure the scenes; consider reducing icon size or opacity.

Ensure high-resolution versions of all figures.

Procedures

Clarify how many participants rated each stimulus.

Provide more detail on the exclusion criteria (e.g., <5 min completion time) and typical completion time.

Specify whether participants used headphones or speakers, as this influences auditory perception.

Statistics

Report effect sizes consistently (e.g., ηp²).

Provide descriptive statistics for naturalness across categories in supplementary material.

Provide ICC confidence intervals for transparency.

4. Conclusion

This manuscript offers a valuable contribution by integrating sensory modalities and exploring restorative perception in tropical environments—an underrepresented area in the literature. The methodology is promising, and the collected stimulus set is impressive. Addressing the methodological and analytical issues outlined above will greatly enhance the manuscript’s rigor, interpretability, and impact.

I encourage the authors to revise the manuscript thoroughly and resubmit, as it has strong potential for publication after substantial improvements.

Reviewer #2: Thank you to the Editor for the opportunity to review the manuscript “Sensing nature in the city: The role of sight and sound in restorative tropical urban green spaces”. The study addresses an important and underexplored topic: the restorative potential of tropical urban green spaces and the role of different sensory modalities. The large and diverse stimulus set, as well as the attention to multisensory perception, are clear strengths. The manuscript is generally well written and accessible.

However, before this work can be considered for publication, major revisions are required. My main concerns relate to theoretical framing, the appropriateness of the statistical design, the validity of the measures used, and the interpretation of findings.

Below, I provide detailed comments and suggestions:

Major Issues

- From a conceptual standpoint, the introduction provides a broad overview of theories and constructs (ART, SRT, naturalness, multisensory integration), but it would benefit from a more focused and theoretically cohesive narrative. At present, the motivation for the study feels somewhat descriptive rather than driven by a clear theoretical gap. It would be helpful to more explicitly articulate why tropical environments might differ from those studied in temperate climates, and how multisensory cues theoretically relate to restorative processes. More importantly, the hypotheses would be stronger if they emerged more directly from this theoretical framing rather than simply reflecting expected differences between scene categories.

- My main concern is that the statistical approach is strongly inappropriate for the study design. Your analysis treats stimuli as the unit of analysis (N = 360), averaging participant ratings for each stimulus and then running a between-subjects 3X3 ANOVA. The main issue is that this is not a 3x3 ANOVA. Participants did not rate all stimuli; thus, their effects cannot be controlled. Your design probably requires a mixed-effects model given the nested nature of your data.

- Cronbach’s α for the perceived restorativeness scores for visual group condition is 0.42, which is far below acceptable psychometric standards.

- Another issue is related to the stimuli categorization. Although the manuscript provides a priori definitions for nature, mixed, and urban scenes, the manipulation check reveals that participants did not perceive a difference between nature and mixed scenes across any modality. This raises conceptual ambiguity: if the two categories are perceptually indistinguishable, then the theoretical distinction underlying your hypotheses becomes unclear. Effectively, the mixed urban–nature stimuli were too similar to the purely natural ones. In most cases, the ‘urban’ component in the mixed scenes was minimal or barely perceptible, which likely contributed to the absence of clear perceptual differences between the two categories.

- The very high correlation between perceived 475 naturalness and restorativeness in the audio group (r = .92) raises the possibility that naturalness ratings and restorative qualities overlap semantically, and that the two measures capture similar constructs. I suggest the authors to acknowledge this limitation in the discussion.

- The interpretation of the interaction between modality and scene type would benefit from a more cautious approach. The result that mixed scenes were perceived as more restorative than purely natural scenes in the visual-only condition is certainly interesting, but the explanation offered (i.e., absence of urban noise enhances visual restorativeness) remains somewhat speculative. There are several other plausible reasons why this pattern might have emerged. For example, the mixed scenes may have simply been more visually coherent or compositionally balanced, or they might have contained greater scenic complexity that viewers found engaging. It is also possible that aesthetic preferences, unrelated to the presence of urban elements, played a role, or that uncontrolled visual features such as lighting, color saturation, or framing influenced these judgments. I encourage you to acknowledge these alternative explanations and to present a more tempered interpretation of this finding.

Minor Issues

- The rationale for limiting clips to 5 seconds could be expanded.

- Please report instructions regarding the use of headphones for audio stimuli.

- Gender imbalance (62 F, 22 M) could be addressed as a limitation.

- Provide information on randomization procedure in Qualtrics.

- Table 2: ICC for fascination in visual-only is extremely low (0.39); discuss implications.

Overall, I think the manuscript has promise and addresses an underrepresented context in restorative environments research. However, major revisions are necessary, particularly concerning statistical methodology, measurement reliability, and theoretical justification.

I encourage the authors to carefully address the issues raised above. Doing so will substantially strengthen the manuscript and its contribution to the literature.

**********

6. PLOS authors have the option to publish the peer review history of their article (what does this mean?). If published, this will include your full peer review and any attached files.

If you choose “no”, your identity will remain anonymous but your review may still be made public.

**Do you want your identity to be public for this peer review?**     For information about this choice, including consent withdrawal, please see our Privacy Policy.

Reviewer #1: **Yes:**    Peddy Caliari

Reviewer #2: No

**********

[NOTE: If reviewer comments were submitted as an attachment file, they will be attached to this email and accessible via the submission site. Please log into your account, locate the manuscript record, and check for the action link "View Attachments". If this link does not appear, there are no attachment files.]

To ensure your figures meet our technical requirements, please review our figure guidelines: https://journals.plos.org/plosone/s/figures

You may also use PLOS’s free figure tool, NAAS, to help you prepare publication quality figures: https://journals.plos.org/plosone/s/figures#loc-tools-for-figure-preparation.

NAAS will assess whether your figures meet our technical requirements by comparing each figure against our figure specifications.

# Author comment: Author response to Decision Letter 1

**DOI:** 10.1371/journal.pone.0351647.r002

9 Feb 2026

Dear Reviewers,

Thank you for the careful and constructive feedback. A major revision in the current manuscript concerns our statistical approach and analysis pipeline. We agree that the original between-subjects ANOVA conducted on stimulus-averaged scores was not well aligned with the study design because participants rated only a subset of stimuli. Accordingly, we have re-analysed the data using linear mixed-effects modelling (LMM) in R, treating trial-level ratings (participants * stimulus) as the unit of analysis and modelling participants and stimuli as random effects. Environmental scenes, modalities, and their interaction were entered as fixed effects. To ensure consistency and reproducibility, we transitioned all analyses from SPSS to R, including descriptive statistics, manipulation checks, reliability analyses, and the power analysis.

These changes resulted in several updates to the Results. First, manipulation checks were re-analysed using an LMM predicting perceived naturalness. The overall pattern remained consistent with the a priori categorisation, except that nature and mixed urban-nature scenes did not differ significantly in perceived naturalness in the audio modality. Second, we estimated the intraclass correlation coefficients directly from the fitted mixed-effects model, instead of aggregating ratings at the stimulus level. Third, internal consistency of the perceived restorativeness composite score increased substantially when computed at the trial level. Finally, although the interaction between environment and modality remained significant, post-hoc tests no longer supported the hypothesis that bimodal presentations of nature scenes yield higher perceived restorativeness than single-modality presentations. We have updated the manuscript accordingly and provide point-by-point responses in the "Response to Reviewers.docx", indicating where revisions were made.

Attachment Submitted filename: Response to Reviewers.docx

# Peer review: Decision Letter 1

**DOI:** 10.1371/journal.pone.0351647.r003

**Authors:** Annalisa Theodorou

25 Mar 2026

PONE-D-25-54884R1

Sensing nature in the city: The role of sight and sound in restorative tropical urban green spaces

PLOS One

Dear Dr. Hoo Ju Yun,

Thank you for submitting your manuscript to PLOS ONE. After careful consideration, we feel that it has merit but does not fully meet PLOS ONE’s publication criteria as it currently stands. Therefore, we invite you to submit a revised version of the manuscript that addresses the points raised during the review process.

==============================

The reviewers and I believe that the manuscript has undergone significant improvement. Nevertheless, some points remain unclearly addressed. Please provide a publicly available dataset through a DOI, following the Data Availability policies of the journal available at https://journals.plos.org/plosone/s/data-availability

In your response letter, please address all the clarifications requested by the reviewer, such as those regarding model specifications, the wording of the restorativeness items, power analysis, and the limitation section.

==============================

Please submit your revised manuscript by May 09 2026 11:59PM. If you will need more time than this to complete your revisions, please reply to this message or contact the journal office at plosone@plos.org. When you're ready to submit your revision, log on to https://www.editorialmanager.com/pone/ and select the 'Submissions Needing Revision' folder to locate your manuscript file.

Please include the following items when submitting your revised manuscript:

- A letter that responds to each point raised by the academic editor and reviewer(s). You should upload this letter as a separate file labeled 'Response to Reviewers'.
- A marked-up copy of your manuscript that highlights changes made to the original version. You should upload this as a separate file labeled 'Revised Manuscript with Track Changes'.
- An unmarked version of your revised paper without tracked changes. You should upload this as a separate file labeled 'Manuscript'.

If you would like to make changes to your financial disclosure, please include your updated statement in your cover letter. Guidelines for resubmitting your figure files are available below the reviewer comments at the end of this letter.

If applicable, we recommend that you deposit your laboratory protocols in protocols.io to enhance the reproducibility of your results. Protocols.io assigns your protocol its own identifier (DOI) so that it can be cited independently in the future. For instructions see: https://journals.plos.org/plosone/s/submission-guidelines#loc-laboratory-protocols. Additionally, PLOS ONE offers an option for publishing peer-reviewed Lab Protocol articles, which describe protocols hosted on protocols.io. Read more information on sharing protocols at https://plos.org/protocols?utm_medium=editorial-email&utm_source=authorletters&utm_campaign=protocols.

As the corresponding author, your ORCID iD is verified in the submission system and will appear in the published article. PLOS supports the use of ORCID, and we encourage all coauthors to register for an ORCID iD and use it as well. Please encourage your coauthors to verify their ORCID iD within the submission system before final acceptance, as unverified ORCID iDs will not appear in the published article. *Only*     the individual author can complete the verification step; PLOS staff *cannot*     verify ORCID iDs on behalf of authors.

We look forward to receiving your revised manuscript.

Kind regards,

Annalisa Theodorou

Academic Editor

PLOS One

Journal Requirements:

If the reviewer comments include a recommendation to cite specific previously published works, please review and evaluate these publications to determine whether they are relevant and should be cited. There is no requirement to cite these works unless the editor has indicated otherwise.

Additional Editor Comments :

[Note: HTML markup is below. Please do not edit.]

Reviewers' comments:

Reviewer's Responses to Questions

**Comments to the Author**

1. If the authors have adequately addressed your comments raised in a previous round of review and you feel that this manuscript is now acceptable for publication, you may indicate that here to bypass the “Comments to the Author” section, enter your conflict of interest statement in the “Confidential to Editor” section, and submit your "Accept" recommendation.

Reviewer #1: (No Response)

Reviewer #2: All comments have been addressed

**********

2. Is the manuscript technically sound, and do the data support the conclusions?

The manuscript must describe a technically sound piece of scientific research with data that supports the conclusions. Experiments must have been conducted rigorously, with appropriate controls, replication, and sample sizes. The conclusions must be drawn appropriately based on the data presented.

Reviewer #1: Yes

Reviewer #2: Yes

**********

3. Has the statistical analysis been performed appropriately and rigorously?

Reviewer #1: Yes

Reviewer #2: Yes

**********

4. Have the authors made all data underlying the findings in their manuscript fully available?

The PLOS Data policy requires authors to make all data underlying the findings described in their manuscript fully available without restriction, with rare exception (please refer to the Data Availability Statement in the manuscript PDF file). The data should be provided as part of the manuscript or its supporting information, or deposited to a public repository. For example, in addition to summary statistics, the data points behind means, medians and variance measures should be available. If there are restrictions on publicly sharing data—e.g. participant privacy or use of data from a third party—those must be specified.

Reviewer #1: No

Reviewer #2: No

**********

5. Is the manuscript presented in an intelligible fashion and written in standard English?

PLOS ONE does not copyedit accepted manuscripts, so the language in submitted articles must be clear, correct, and unambiguous. Any typographical or grammatical errors should be corrected at revision, so please note any specific errors here.

Reviewer #1: Yes

Reviewer #2: Yes

**********

6. Review Comments to the Author

Please use the space provided to explain your answers to the questions above. You may also include additional comments for the author, including concerns about dual publication, research ethics, or publication ethics. (Please upload your review as an attachment if it exceeds 20,000 characters)

Reviewer #1: Thank you for the substantial revision. The shift from stimulus-averaged ANOVA to trial-level cross-classified linear mixed-effects models (participants × stimuli) directly addresses the main design–analysis misalignment and markedly improves the technical soundness and reproducibility of the work. I also appreciate that you updated conclusions when some original post-hoc patterns (e.g., a hypothesized bimodal advantage for nature scenes) were no longer supported after reanalysis. Overall, the manuscript is close to publishable.

Essential point (PLOS ONE compliance): Data, code, and stimulus mapping must be verifiably available

At present, I could not verify that a full reproducibility package is publicly available via a stable repository link/DOI (or as complete Supporting Information). Before acceptance, please provide: (i) the trial-level dataset (participant ID anonymized, stimulus ID, modality, environment category, naturalness, the 5 restorativeness items, and any exclusions), (ii) a codebook/metadata file, (iii) the analysis scripts (R code) required to reproduce all results/figures/tables, and (iv) a stimulus ID mapping file linking each stimulus ID to the corresponding audio/visual/bimodal files and the a priori scene categorization. If media files cannot be shared publicly (e.g., licensing/privacy), please state exactly what cannot be shared and why, and provide an ethics-approved access route consistent with PLOS policy. “Available upon request” would not be sufficient.

Minor but important clarifications

Please report the LMM specification transparently (exact model formulae; df method such as Kenward–Roger/Satterthwaite; brief diagnostics/convergence; whether random slopes were explored and, if not, why).

Because the audio condition uses adapted soundscape wording for the restorativeness items, please strengthen justification for cross-modality comparability and/or temper interpretation accordingly (a brief sensitivity check would be welcome).

Please clarify how the updated power analysis was performed in the LMM framework (ideally simulation-based), ensuring it matches the inferential approach.

Consider consolidating limitations in a clearly labeled paragraph (online setting; unstandardized audio playback; brief exposure duration; sample composition; geographic generalizability).

The reanalysis is technically sound and the manuscript is close to publishable. However, I could not verify that the underlying trial-level dataset, analysis code, and stimulus ID mapping are publicly available via a stable repository link/DOI or as complete Supporting Information, as required by PLOS ONE’s data policy. Please provide a verifiable reproducibility package prior to acceptance.

Reviewer #2: (No Response)

**********

7. PLOS authors have the option to publish the peer review history of their article (what does this mean?). If published, this will include your full peer review and any attached files.

If you choose “no”, your identity will remain anonymous but your review may still be made public.

**Do you want your identity to be public for this peer review?**     For information about this choice, including consent withdrawal, please see our Privacy Policy.

Reviewer #1: **Yes:**    Peddy Caliari

Reviewer #2: **Yes:**    Silvana Mula

**********

[NOTE: If reviewer comments were submitted as an attachment file, they will be attached to this email and accessible via the submission site. Please log into your account, locate the manuscript record, and check for the action link "View Attachments". If this link does not appear, there are no attachment files.]

To ensure your figures meet our technical requirements, please review our figure guidelines: https://journals.plos.org/plosone/s/figures

You may also use PLOS’s free figure tool, NAAS, to help you prepare publication quality figures: https://journals.plos.org/plosone/s/figures#loc-tools-for-figure-preparation.

NAAS will assess whether your figures meet our technical requirements by comparing each figure against our figure specifications.

# Author comment: Author response to Decision Letter 2

**DOI:** 10.1371/journal.pone.0351647.r004

15 Apr 2026

We thank the Reviewers for the thorough and constructive feedback on our revised manuscript. We are pleased that the Reviewer found the shift to trial-level cross-classified linear mixed-effects model to be a substantial improvement and that the manuscript is considered close to be publishable. Below, we address each comment in detail.

Response to Reviewer 1

1. Thank you for highlighting this important requirement. We have now deposited a complete reproducibility package on the Open Science Framework (OSF). The repository is organised into two components to separate stimulus materials from study-specific data and analysis files.

Main project: Tropical Urban Green Space Restorativeness Project

DOI: https://doi.org/10.17605/OSF.IO/4CBYQ

Component 1 - Tropical Environmental Stimuli Database (TESD)

- Stimulus files: The complete set of 120 environmental stimuli (audio, visual, and bimodal formats) used in the study.

- Stimulus mapping file (Stimulus_Mapping.csv): links each stimulus ID to its corresponding media filenames and a priori environment categorisation.

Component 2 – Sensing Nature In The City: The Role of Sight and Sound in Tropical Urban Green Spaces

- Trial-level dataset (long_clean_dataset_with_PRS.xlsx): Contains anonymised participant IDs, stimulus IDs, modality condition, environment category, naturalness ratings, the five restorative items, and the composite perceived restorativeness score. Excluded participants (n = 9) are not included in the dataset; exclusion criteria are described in the manuscript (Section 2.5).

- Codebook (Codebook.md): Describes each variable, including measurement scales, factor levels, and data processing steps.

- Analysis scripts (LMM_Latest.Rmd): Contains all R code required to reproduce the reported results, figures, and tables.

2. Thank you for this suggestion. We have revised the Analysis Plan (Section 2.6) to report the LMM specification more transparently. The revised text now includes:

a. Exact model formula: The exact model formula is stated in Line 359 –360.

b. Degrees of freedom method: Omnibus F-tests used Satterthwaite approximations for denominator degrees of freedom and p-values. Post-hoc pairwise comparisons used Kenward-Roger degrees of freedom with Bonferroni correction. (Line 366 – 367).

c. Convergence and diagnostics: All models converged without warnings. Inspection of scaled residuals indicated no substantial departures from model assumptions. (Line 360 –361)

d. Random slopes: Random slopes for modality could not be estimated because participants were nested within modality conditions (each participant rated stimuli in only one modality). Random slopes for environment were explored but resulted in singular fits, indicating insufficient variance to support their estimation. (Line 361 – 364).

3. Thank you for flagging this up. We have strengthened the justification for cross-modality comparability in the Measures section (Line 290 – 294). Specifically, we have added text clarifying that although the PR-SF and PRS-SF differ in surface-level wording to reflect their respective modalities, both scales operationalise the same five restorative components drawn from ART. This adaptation follows the same rationale as Uebel et al. (2021), whom we cited in the original manuscript. Given this conceptual alignment (grounded in shared theoretical structure and a precedent in the literature) and the item-level correspondence across scales, we consider the measures sufficiently comparable for the purposes of the present analyses. Nonetheless, as a matter of methodological transparency, we acknowledge in the Discussion (Line 586 – 590) that modality-specific wording may introduce measurement variance that limits the interpretability of direct cross-modality comparisons.

4. Thank you for this suggestion. We have revised the power analysis description in Section 2.5 (Line 336 – 339) to clarify that simulation-based methods were used. Specifically, power was estimated using the “simr” package in R, which generates Monte Carlo simulations from the fitted cross-classified LMM. The Environment × Modality interaction was tested using a likelihood ratio test, matching the inferential approach used in the main analyses.

5. Thank you for this suggestion. We have consolidated the limitations into the final paragraphs of the Discussion (Lines 581 – 609), addressing methodological considerations (online setting, unstandardised audio playback, brief exposure duration, modality-specific measures) as well as limitations relating to stimuli and sample characteristics. We have also reframed the future research directions positively, emphasising how subsequent studies can build on the present findings.

Attachment Submitted filename: Response to Reviewers Letter.docx

# Peer review: Decision Letter 2

**DOI:** 10.1371/journal.pone.0351647.r005

**Authors:** Yuting YIN

1 Jun 2026

Sensing nature in the city: The role of sight and sound in restorative tropical urban green spaces

PONE-D-25-54884R2

Dear Dr. Yun,

We’re pleased to inform you that your manuscript has been judged scientifically suitable for publication and will be formally accepted for publication once it meets all outstanding technical requirements.

Within one week, you’ll receive an e-mail detailing the required amendments. When these (and comments from the reviewers listed below) have been addressed, you’ll receive a formal acceptance letter and your manuscript will be scheduled for publication.

An invoice will be generated when your article is formally accepted. Please note, if your institution has a publishing partnership with PLOS and your article meets the relevant criteria, all or part of your publication costs will be covered. Please make sure your user information is up-to-date by logging into Editorial Manager at Editorial Manager® and clicking the ‘Update My Information' link at the top of the page. For questions related to billing, please contact billing support.

If your institution or institutions have a press office, please notify them about your upcoming paper to help maximize its impact. If they’ll be preparing press materials, please inform our press team as soon as possible -- no later than 48 hours after receiving the formal acceptance. Your manuscript will remain under strict press embargo until 2 pm Eastern Time on the date of publication. For more information, please contact onepress@plos.org.

Kind regards,

Yuting YIN

Academic Editor

PLOS One

Additional Editor Comments (optional):

Reviewers' comments:

Reviewer's Responses to Questions

**Comments to the Author**

1. If the authors have adequately addressed your comments raised in a previous round of review and you feel that this manuscript is now acceptable for publication, you may indicate that here to bypass the “Comments to the Author” section, enter your conflict of interest statement in the “Confidential to Editor” section, and submit your "Accept" recommendation.

Reviewer #1: All comments have been addressed

Reviewer #3: All comments have been addressed

**********

2. Is the manuscript technically sound, and do the data support the conclusions?

The manuscript must describe a technically sound piece of scientific research with data that supports the conclusions. Experiments must have been conducted rigorously, with appropriate controls, replication, and sample sizes. The conclusions must be drawn appropriately based on the data presented.

Reviewer #1: Yes

Reviewer #3: Yes

**********

3. Has the statistical analysis been performed appropriately and rigorously?

Reviewer #1: Yes

Reviewer #3: I Don't Know

**********

4. Have the authors made all data underlying the findings in their manuscript fully available?

The PLOS Data policy requires authors to make all data underlying the findings described in their manuscript fully available without restriction, with rare exception (please refer to the Data Availability Statement in the manuscript PDF file). The data should be provided as part of the manuscript or its supporting information, or deposited to a public repository. For example, in addition to summary statistics, the data points behind means, medians and variance measures should be available. If there are restrictions on publicly sharing data—e.g. participant privacy or use of data from a third party—those must be specified.

Reviewer #1: Yes

Reviewer #3: Yes

**********

5. Is the manuscript presented in an intelligible fashion and written in standard English?

PLOS ONE does not copyedit accepted manuscripts, so the language in submitted articles must be clear, correct, and unambiguous. Any typographical or grammatical errors should be corrected at revision, so please note any specific errors here.

Reviewer #1: Yes

Reviewer #3: Yes

**********

6. Review Comments to the Author

Please use the space provided to explain your answers to the questions above. You may also include additional comments for the author, including concerns about dual publication, research ethics, or publication ethics. (Please upload your review as an attachment if it exceeds 20,000 characters)

Reviewer #1: Thank you for the thorough and substantive revision. The shift from stimulus-averaged ANOVA to trial-level cross-classified linear mixed-effects models (participants × stimuli) is now well aligned with the study design, and the statistical reporting is clearer (model specification, df methods, post-hoc corrections, and diagnostics). I also appreciate that conclusions were updated where earlier post-hoc patterns were no longer supported after reanalysis.

Open science / reproducibility: Thank you for depositing a complete reproducibility package on OSF (including the full stimulus set, a stimulus mapping file, the trial-level dataset, a codebook, and analysis scripts). This addresses the key PLOS ONE data policy requirement. Please ensure the final manuscript and submission metadata consistently reference the OSF DOI and that file names/paths correspond exactly to those cited (mapping, dataset, scripts).

Minor points for final polishing:

Please do a final proofread for small grammatical/typographical issues (a few tense inconsistencies remain in the Procedure section).

Consider keeping a short, explicit sentence in the Discussion emphasizing that 5-second clips capture rapid appraisals rather than sustained restoration (already noted as a limitation; retaining it prominently is helpful).

Overall, the manuscript is technically sound and close to publishable.

Reviewer #3: Dear Authors,

Thank you for giving me the opportunity to review your article, which addresses a highly topical issue. The paper is well-structured and clearly written. While it does not present groundbreaking new findings, this is not a strict criterion for this journal.

In your discussion, you mention as a limitation that men and women may perceive things differently. I completely agree with you on this point. However, why did you not compare the responses of male and female participants? Do they actually differ in their perceptions within your study? Conducting this comparison could yield some highly interesting insights. Could you please supplement the paper with this analysis?

Thank you.

**********

7. PLOS authors have the option to publish the peer review history of their article (what does this mean?). If published, this will include your full peer review and any attached files.

If you choose “no”, your identity will remain anonymous but your review may still be made public.

**Do you want your identity to be public for this peer review?**     For information about this choice, including consent withdrawal, please see our Privacy Policy.

Reviewer #1: **Yes:**    Peddy Caliari

Reviewer #3: No

**********

# Editor report: Acceptance letter

**DOI:** 10.1371/journal.pone.0351647.r006

**Authors:** Yuting YIN

PONE-D-25-54884R2

PLOS One

Dear Dr. Hoo Ju Yun,

I'm pleased to inform you that your manuscript has been deemed suitable for publication in PLOS One. Congratulations! Your manuscript is now being handed over to our production team.

At this stage, our production department will prepare your paper for publication. This includes ensuring the following:

* All references, tables, and figures are properly cited

* All relevant supporting information is included in the manuscript submission,

* There are no issues that prevent the paper from being properly typeset

You will receive further instructions from the production team, including instructions on how to review your proof when it is ready. Please keep in mind that we are working through a large volume of accepted articles, so please give us a few days to review your paper and let you know the next and final steps.

Lastly, if your institution or institutions have a press office, please let them know about your upcoming paper now to help maximize its impact. If they'll be preparing press materials, please inform our press team within the next 48 hours. Your manuscript will remain under strict press embargo until 2 pm Eastern Time on the date of publication. For more information, please contact onepress@plos.org.

You will receive an invoice from PLOS for your publication fee after your manuscript has reached the completed accept phase. If you receive an email requesting payment before acceptance or for any other service, this may be a phishing scheme. Learn how to identify phishing emails and protect your accounts at https://explore.plos.org/phishing.

If we can help with anything else, please email us at customercare@plos.org.

Thank you for submitting your work to PLOS ONE and supporting open access.

Kind regards,

PLOS ONE Editorial Office Staff

on behalf of

Dr. Yuting YIN

Academic Editor

PLOS One
